# A systematic review and meta-analysis of inflammatory biomarkers in Parkinson’s disease

**DOI:** 10.1038/s41531-023-00449-5

**Published:** 2023-02-04

**Authors:** Yi Qu, Jiangting Li, Qixiong Qin, Danlei Wang, Jingwei Zhao, Ke An, Zhijuan Mao, Zhe Min, Yongjie Xiong, Jingyi Li, Zheng Xue

**Affiliations:** Department of Neurology, Tongji Hospital, Tongji Medical College, Huazhong University of Science and Technology, Wuhan, China

**Keywords:** Parkinson's disease, Diagnostic markers

## Abstract

Neuroinflammation plays a crucial role in the pathogenesis of Parkinson’s disease (PD), but controversies persist. Studies reporting concentrations of blood or cerebrospinal fluid (CSF) markers for patients with PD and controls were included and extracted. Pooled Hedges’g was adopted to illustrate comparisons, and covariates were used to explore sources of heterogeneity. Finally, 152 studies were included. Increased IL-6, TNF-α, IL-1β, STNFR1, CRP, CCL2, CX3CL1, and CXCL12 levels and decreased INF-γ and IL-4 levels were noted in the PD group. In addition, increased CSF levels of IL-6, TNF-α, IL-1β, CRP and CCL2 were revealed in patients with PD compared to controls. Consequently, significantly altered levels of inflammatory markers were verified between PD group and control, suggesting that PD is accompanied by inflammatory responses in both the peripheral blood and CSF. This study was registered with PROSPERO, CRD42022349182.

## Introduction

Parkinson’s disease (PD) is the second most common neurodegenerative diseases, which exhibits diverse clinical features including motor and nonmotor symptoms^1^, and leads to decreased quality of daily life, disability or eventually death in the elderly^2^. PD is characterized by the selective loss of dopaminergic neurons in the substantia nigra (SN) pars compacta, but the exact aetiology remains unclear^3^. Increasing evidence has suggested that central and peripheral inflammation play vital roles in the pathologic features and symptoms of PD^4^, and several peripheral biomarkers exhibit tracing and detection accuracy for disease severity and progression^4,5^.

Varieties inflammatory markers, including cytokines such as the interleukin (IL) and tumour necrosis factor (TNF); chemokines such as chemokine ligand (CCL) and CX3 chemokine ligand (CX3CL); and the acute phase reactant protein C-reactive protein (CRP), have been reported as critical signalling molecules of immune activation that exert effects in the central nervous system (CNS) and periphery^6^. In addition, peripheral inflammation can contribute to the aetiology and progress of PD^7^. The less invasive markers present in peripheral blood and cerebrospinal fluid (CSF) can assist in better understanding the aetiology of PD and provide candidate biomarkers for the disease; however, their performances varies greatly in different studies due to differences in research sites and tools.

Previous reviews and meta-analyses have demonstrated that the levels of inflammatory markers in the peripheral blood and CSF of patients with PD differ from those for healthy populations^8–10^. However, some of these markers lack quantitative analyses, recent updated information, or comprehensive included inflammatory markers. To explore the real altered levels of each marker, this meta-analysis and systematic review aimed to verify whether the concentrations of specific inflammatory markers in peripheral blood and CSF differ quantitatively between patients with PD and normal controls.

## Results

**Fig. 1:**
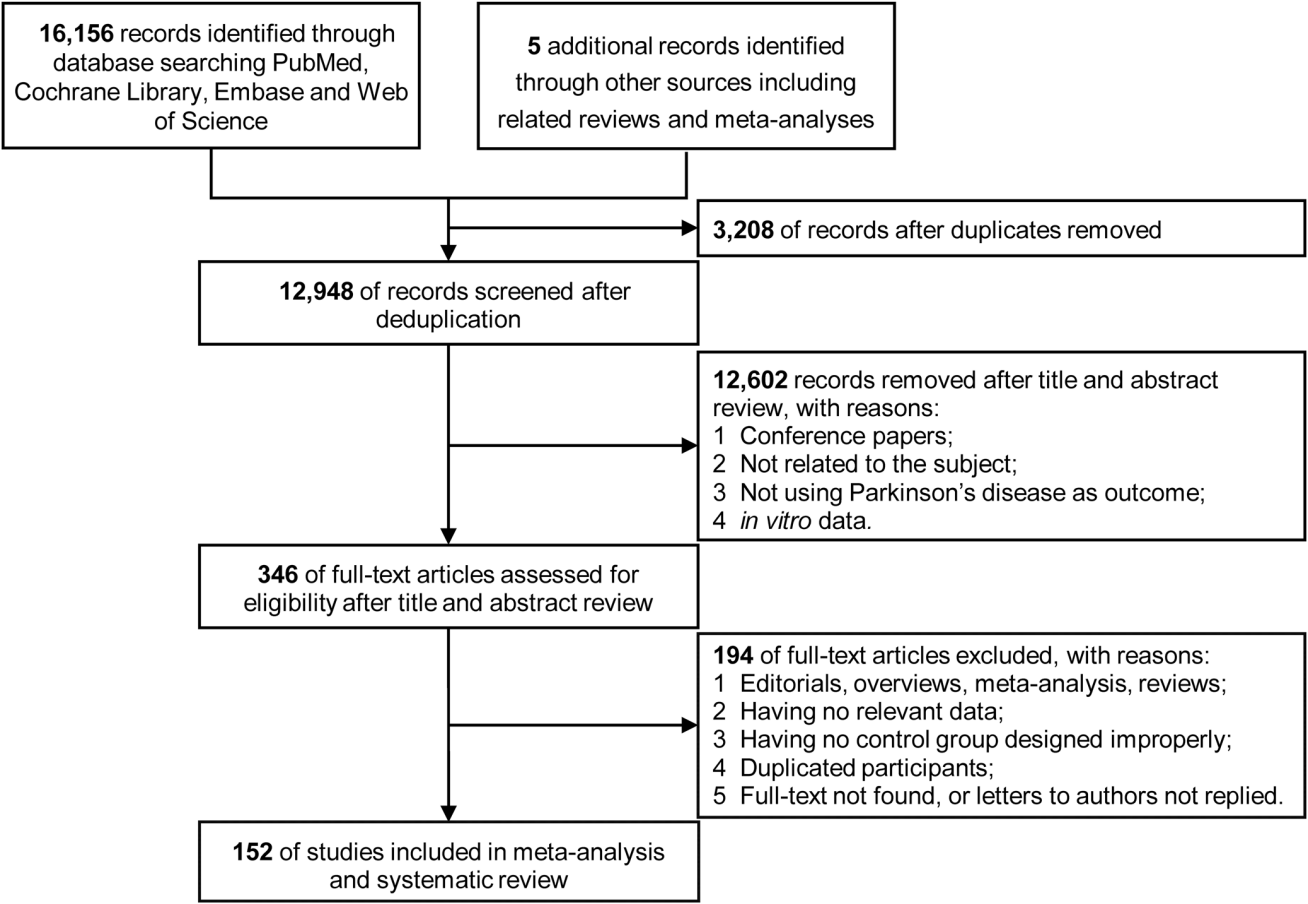
Flowchart of study inclusion and exclusion. A total of 16,161 records were identified. After literature searching, selection and deduplication, 152 studies measuring peripheral blood or CSF inflammatory markers were finally included in the systematic reviews and meta-analyses.

A total of 16,156 records were identified after literature searching, selection and deduplication, and 152 studies measuring peripheral blood or CSF inflammatory markers were finally included in the systematic reviews and meta-analyses (Fig. 1). The characteristics and quality assessments are listed in Supplementary Table 1–2 which encompassed 9,032 patients diagnosed with PD and 12,628 controls. In total, 92 markers were analysed, and the official marker names are presented in Supplementary Table 3. Performances and heterogeneity analyses of individual markers are shown in Supplementary Tables 4-5.

### Comparisons of peripheral blood biomarkers between PD patients and control

**Fig. 2:**
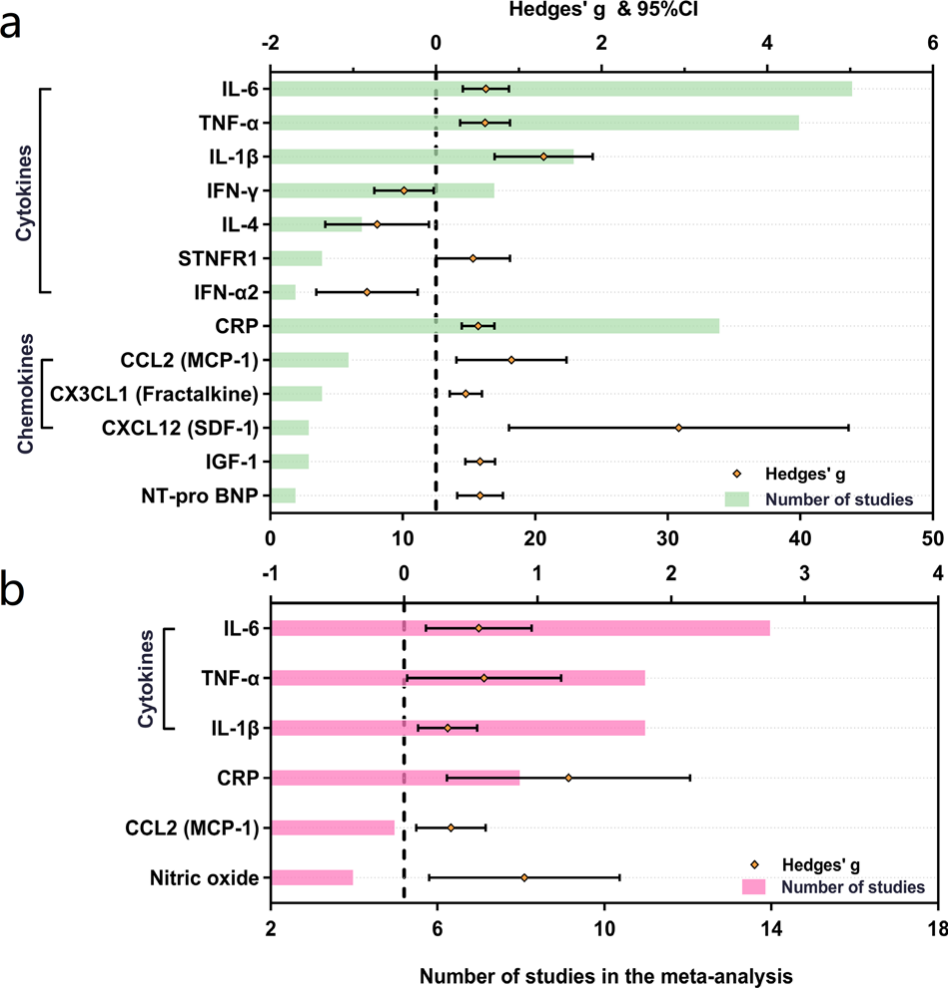
Comparative outcomes of peripheral blood and cerebrospinal fluid biomarkers in the meta-analysis. The peripheral blood (**a**) and cerebrospinal fluid (**b**) inflammatory markers with significant effect sizes (Hedges’ g) were displayed in comparisons for PD patients versus controls. Orange spots indicate Hedges’ g of each marker, and green and pink bars indicate the number of studies included. CCL chemokine (C-C motif) ligand, CRP C-reactive protein, CX3CL CX3 chemokine ligand, CXCL chemokine (C-X-C motif) ligand, IFN Interferon, IL interleukin, MCP monocyte chemoattractant protein, NT-pro BNP N-terminal pro-B-type natriuretic peptide, PD Parkinson’s disease, SDF stromal cell-derived factor, STNFR soluble tumour necrosis factor receptor, TNF tumour necrosis factor.

**Fig. 3:**
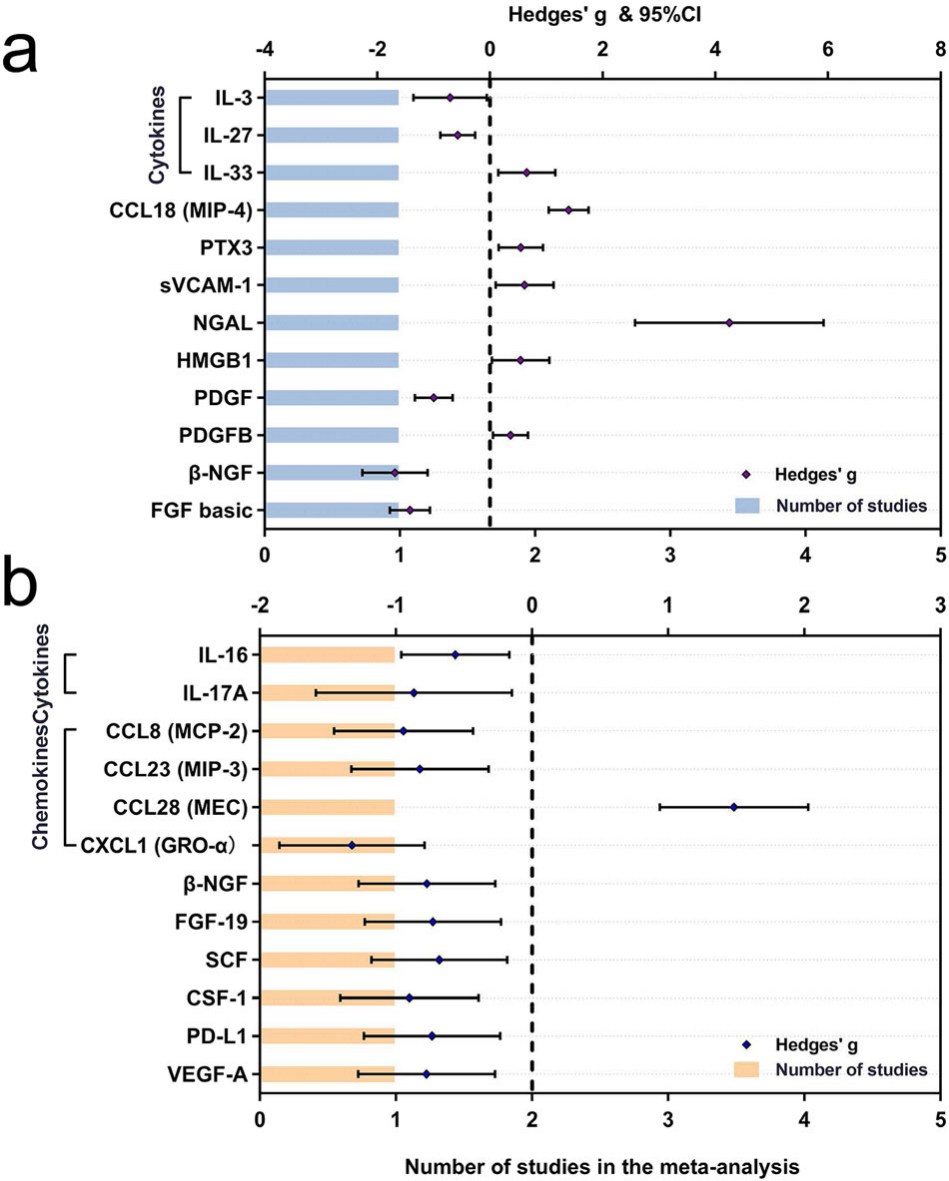
Comparative outcomes of peripheral blood and cerebrospinal fluid biomarkers in the systematic review. The peripheral blood (**a**) and cerebrospinal fluid (**b**) inflammatory markers with significant effect sizes (Hedges’ g) were displayed in comparisons for PD patients versus controls. Violet spots indicate Hedges’ g of each marker, and blue and orange bars indicate the number of studies included. CCL chemokine (C-C motif) ligand, CSF macrophage-colony stimulating factor, CXCL chemokine (C-X-C motif) ligand, FGF fibroblast growth factor, GRO growth-regulated oncogene, IL interleukin, HMGB high-mobility group box, MCP monocyte chemoattractant protein, MEC mucosae-associated epithelial chemokine, MIP macrophage inflammatory protein, NGAL neutrophil gelatinase-associated lipocalin, NGF nerve growth factor, PD Parkinson’s disease, PDGFB platelet-derived growth factor-B, PTX pentraxin, PD-L programmed death-ligand, SCF stem cell factor, SDF stromal cell-derived factor, sVCAM soluble vascular cell adhesion molecule, VEGF-A vascular endothelial growth factor A.

Random-effects results demonstrated that patients with PD had higher peripheral blood levels of IL-6 (Hedges’ g 0.603; 95%CI 0.325 to 0.881, *P* < 0.001), TNF-α (Hedges’ g 0.593; 95%CI 0.293 to 0.894, *P* < 0.001), IL-1β (Hedges’ g 1.300; 95%CI 0.709 to 1.892, *P* < 0.001), soluble TNF receptor 1 (sTNFR1; Hedges’ g 0.449; 95%CI 0.004 to 0.894, *P* = 0.048), CRP (Hedges’ g 0.510; 95%CI 0.313 to 0.706, *P* < 0.001), CCL2 (Hedges’ g 0.911; 95%CI 0.246 to 1.576, *P* = 0.007), CX3CL1 (Hedges’ g 0.361; 95%CI 0.166 to 0.556, *P* < 0.001), CX chemokine ligand 12 (CXCL12; Hedges’ g 2.933; 95%CI 0.883 to 4.983, *P* = 0.005), insulin-like growth factor-1 (IGF-1; Hedges’ g 0.534; 95%CI 0.355 to 0.714, *P* < 0.001) and N-terminal pro-B-type natriuretic peptide (NT-pro BNP; Hedges’ g 0.533; 95%CI 0.256 to 0.809, *P* < 0.001). Furthermore, significantly decreasing concentrations were revealed for IFN-γ (Hedges’ g -0.385; 95%CI -0.743 to -0.026, *P* = 0.035), IL-4 (Hedges’ g -0.710; 95%CI -1.336 to -0.084, *P* = 0.026) and IFN-α2 (Hedges’ g -0.831; 95%CI -1.444 to -0.219, *P* = 0.008) (Fig. 2a). Then, the systematic review identified some underlying inflammatory markers reported in one study that were significantly changed in patients with PD, including elevated levels of IL-33, CCL18, Pentraxin 3 (PTX3), soluble vascular cell adhesion molecule-1(sVCAM-1), neutrophil gelatinase-associated lipocalin (NGAL), high mobility group 1 (HMGB1) and platelet-derived growth factor-B (PDGFB), as well as reduced levels of IL-3, IL-27, PDGF, β-nerve growth factor (NGF) and fibroblast growth factor (FGF)-basic (Fig. 3a). Other blood biomarkers that were altered in PD group are presented in Supplementary Figs. 1–2.

### Comparisons of CSF biomarkers between PD patients and control

Random-effects meta-analyses also showed increased CSF levels of IL-6 (Hedges’ g 0.559; 95%CI 0.163 to 0.955, *P* = 0.006), TNF-α (Hedges’ g 0.599; 95%CI 0.023 to 1.175, *P* = 0.024), IL-1β (Hedges’ g 0.326; 95%CI 0.105 to 0.547, *P* = 0.004), CRP (Hedges’ g 1.231; 95%CI 0.321 to 2.141, *P* = 0.008), CCL2 (Hedges’ g 0.351; 95%CI 0.090 to 0.612, *P* = 0.008) and nitric oxide (NO; Hedges’ g 0.901; 95%CI 0.188 to 1.614, *P* = 0.013) (Fig. 2b). Moreover, lower concentrations of IL-16, IL-17A, CCL8, CCL23, CXCL1, β-NGF, FGF-19, stem cell factor (SCF), macrophage-colony stimulating factor (CSF-1), programmed death-ligand 1 (PD-L1) and vascular endothelial growth factor A (VEGF-A) were discovered in PD participants than in controls, whereas increased levels of CCL28 were detected. The nonsignificant markers in CSF for patients with PD were shown in Supplementary Figs. 3–4.

### Publication biases and sensitivity analyses

**Fig. 4:**
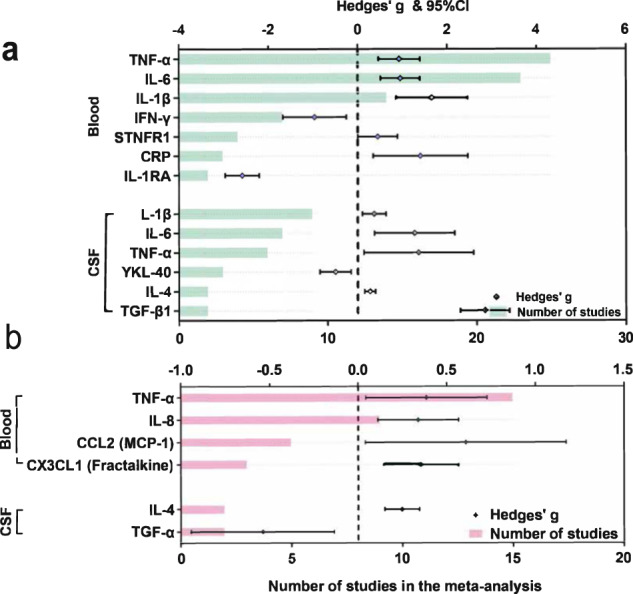
Subgroup comparative outcomes of fluid biomarkers stratified by different assay types in the meta-analysis. Significant comparisons of peripheral blood and CSF biomarkers using ELISA (**a**) and multiplex cytokine (**b**) are shown. Inflammatory markers with significant effect sizes (Hedges’ g) were displayed in comparisons of PD patients versus controls. Violet (**a**) and dark green (**b**) spots indicate Hedges’ g of each marker, and green (**a**) and pink (**b**) bars indicate the number of studies included. *Abbreviations*: CRP C-reactive protein, CSF cerebrospinal fluid, CX3CL CX3 chemokine ligand, ELISA enzyme-linked immunosorbent assay; IFN interferon, IL interleukin, IL-1RA IL-1 receptor antagonist, MCP monocyte chemoattractant protein, TGF transforming growth factor, TNF tumour necrosis factor, YKL chitinase-3-like protein.

Egger’s tests identified that publication biases were found for IL-6, CRP, IL-1β, IFN-γ and STNFR1 in peripheral blood (*P* < 0.050), as well as IL-6, TNF-α and NO in CSF. These findings suggested the data for these markers were not sufficiently robust. The conflicting findings among studies might be due to differences in assays used to detect cytokines and chemokines, such as conventional enzyme-linked immunosorbent assay (ELISA), multiplex cytokine panel and cytometric beads array (CBA). Then, the sensitivity analyses were employed to reduce these biases and subgroup analyses were performed according to assay types. On the one hand, random-effects meta-analyses showed that increased levels of TNF-α, IL-6, IL-1β, STNFR1and CRP among PD patients were identified in peripheral blood. The increased concentrations of IL-1β, IL-6, TNF-α, IL-4 and transforming growth factor (TGF)-β in CSF were identified using ELISA. Similarly, reduced levels of IFN-γ and IL-1 receptor antagonist (IL-1RA) in peripheral blood, as well as chitinase protein 40 (YKL-40) in CSF were observed (Fig. 4a). On the other hand, TNF-α, IL-8, CCL2 and CX3CL1 in blood were significantly elevated in subjects with PD as determined using multiplex panels. Additionally, increased IL-4 and decreased TGF-α levels were detected in CSF (Fig. 4b).

### Diagnostic accuracy of inflammatory biomarkers in the identification of PD

Single and combined markers of inflammation were used in the systematic review of 17 and 7 eligible studies, respectively. On the one hand, more than one study illustrated that CRP in peripheral blood, as well as soluble triggering receptor expressed on myeloid cells 2 (sTREM2), central nervous system specific protein beta (S100β) and YKL-40 in CSF, exhibited good diagnostic accuracy in distinguishing PD patients from controls. In addition, sVCAM-1, NOD-like receptor thermal protein domain associated protein 3 (NLRP3), IL-1β, CXCL12 and IL-8 in blood showed excellent diagnostic values (area under the curve [AUC] > 0.80), whereas PTX3, serum amyloid A (SAA) and CX3CL1 in blood, as well as amyloid precursor protein-alpha (sAPP-α), TNF-α and IL-6 showed moderate accuracy (AUC 0.60-0.80). On the other hand, the sensitivity and specificity of a single biomarker were insufficient based on the use of the reported assays, and the diagnostic accuracy was greatly enhanced upon the combined use of multiple markers. Specifically, inflammatory markers combined with α-synuclein, AD core biomarkers and basic characteristics yielded optimum values (Table 1).

**Table 1:** The systematic review of the diagnostic accuracy for inflammatory markers.

No.	Author	Year	Sample source	Assay type	Samples		Marker	AUC	Sensitivity	Specificity	Cutoff values	Unit	Summary
					PD	HC							
**Single inflammatory markers**													
1	Lee, H. W.	2011	Plasma	ELISA	66	41	PTX3	0.642 (0.54–0.75)	0.758	0.390	5.415	pg/mg	Plasma PTX3 levels could be a new biochemical marker for PD.
2	Sathe, K.	2012	CSF	ELISA	82	64	S100β	0.76	NA	NA	NA	NA	The ROC curve indicated a moderate discriminative effect.
3	Bartl, M.	2021	CSF	ELISA	252	115	S100β	0.544	NA	NA	NA	NA	The biomarker did not differentiate between PD and controls.
4	Olsson, B.	2013	CSF	ELISA	50	37	YKL-40	NA	0.605	0.815	126368	ng/L	CSF levels of YKL-40 were lower in patients who had PD compared with controls.
5	Zhao, Y.	2022	Plasma	ELISA	36	36	YKL-40	0.72 (0.60–0.84)	NA	NA	NA	NA	YKL-40 was implicated in PD pathogenesis.
6	Bartl, M.	2021	CSF	ELISA	252	115	YKL-40	0.565	NA	NA	NA	NA	The biomarker did not differentiate between PD and controls.
7	Magdalinou, N. K.	2015	CSF	MSD	20	15	sAPPα	NA	0.740	0.650	485	ng/mL	The decreasing levels of sAPPα in PD could be used as markers of disease progression.
8	Delgado-Alvarado, M.	2017	CSF	Luminex Xmap	40	40	TNF-α	0.66 (0.55–0.82)	NA	NA	NA	NA	The CSF TNF-α might serve as biomarkers to diagnose PD.
9	Solmaz, V.	2018	Blood	Nephelometric	101	60	CRP	0.683	0.650	0.700	8.7	mg/L	CRP levels was very important indicators of peripheral inflammation in PD.
10	Baran, A.	2019	Serum	Nephelometric	30	30	CRP	0.70 (0.56–0.86)	0.667	0.777	0.63	mg/L	CRP might be fair markers in the diagnosis of PD.
11	Jin, H.	2020	Serum	NA	183	89	CRP	0.91 (0.87–0.94)	0.749	0.997	5.8	mg/mL	CRP exhibited high sensitivity and specificity for predicting PD.
12	Yang, W. L.	2020	Plasma	Nephelometric	204	204	CRP	0.56 (0.51–0.62)	0.299	0.882	3.05	mg/L	Higher CRP levels might be important markers to assess the PD severity.
13	Perner, C.	2019	Plasma	ELISA	33	33	sVCAM-1	0.96	0.880	0.910	919	ng/mL	Plasma levels of the sVCAM1 were highly increased in patients with PD.
14	Chatterjee, K.	2020	Serum	ELISA	27	15	NLRP3	0.96	NA	NA	NA	NA	Significant serum NLRP3 and IL-1β increment in PD provided evidence for peripheral inflammasome activation.
							IL-1β	0.94	NA	NA	NA	NA	
15	Peng, G.	2020	CSF	ELISA	55	40	sTREM2	0.70 (0.59–0.81)	0.705	0.659	NA	NA	CSF sTREM2 could serve as a promising biomarker.
			Serum				sTREM2	0.55 (0.43–0.67)	0.300	0.878	NA	NA	
16	Bartl, M.	2021	CSF	ELISA	252	115	sTREM2	0.538	NA	NA	NA	NA	The biomarker did not differentiate between PD and controls.
17	Mo, M. S.	2021	CSF	ELISA	80	65	sTREM2	0.79 (0.71–0.87)	NA	NA	NA	NA	CSF sTREM2 might be a potential biomarker for PD.
18	Bartl, M.	2021	CSF	ELISA	252	115	IL-6	0.525	NA	NA	NA	NA	The biomarker did not differentiate between PD and controls.
19	Wu, Z. B.	2021	Serum	NA	58	60	SAA	0.74 (0.66–0.83)	0.638	0.750	NA	NA	The levels of SAA were higher in the PD patients than those of the control group.
20	Li, Y. Y.	2022	Plasma	MSD	76	76	CXCL12	0.83	0.829	0.658	1051	pg/mL	Increased levels of CXCL12, CX3CL1 and IL-8 were independent diagnostic biomarkers of PD.
							CX3CL1	0.63	0.645	0.632	3966.9	pg/mL	
							IL-8	0.85	0.737	0.855	1.7	pg/mL	
**Multiple inflammatory markers**													
1	Delgado-Alvarado, M.	2017	CSF/Plasma	Luminex Xmap	39	38	3 markers	0.92 (0.84–0.99)	0.929	0.750	NA	NA	P-Tau/α-synuclein and TNF-α
2	Dos Santos, M. C. T.	2018	CSF	Millipore	80	80	16 markers	0.71	0.900	0.500	NA	NA	Aβ40, Aβ42, α-synuclein, P-Tau, T-Tau, OPN, NFL, IL-6, DJ-1, UCHL1, FLT3LG, MMP-2, S100β, ApoA1, Aβ40/Aβ42 and p-Tau/t-Tau
							4 markers	0.77	0.850	0.750	NA	NA	S100β, α-synuclein, MMP-2 and UCHL1
3	Calvani, R.	2020	Serum	Luminex Xmap	20	30	7 markers	close to 1	NA	NA	NA	NA	Citrulline, Phosphoethanolamine, Proline, IL-8, IL-9, MIP-1α, MIP-1β
4	Majbour, N. K.	2020	CSF	Luminex Xmap	60	43	5 markers	0.88 (0.81-0.96)	NA	NA	NA	NA	t-, o- and pS129-α-syn, TNF-α, IL-16
5	Yang, W. L.	2020	Plasma	Nephelometric	204	204	5 markers	0.69 (0.64–0.74)	0.475	0.843	NA	NA	SOD, cholesterol, HDL-C, LDL-C, CRP
6	Chen, S. J.	2021	Plasma	ELISA	248	149	4 markers	0.67 (0.62–0.73)	NA	NA	NA	NA	Age, sex, LBP, TNF-α, IL-6 and IL-17A
7	Li, Y. Y.	2022	Plasma	MSD	76	76	4 markers	0.89 (0.84–0.94)	NA	NA	NA	NA	CXCL12, CX3CL1, IL-8 and CCL15

*CXCL12* C-X-C motif ligand 12 protein, *CX3CL1* CX3 chemokine ligand 1, *CRP* C-reactive protein, CSF cerebrospinal fluid, *ELISA* enzyme-linked immunosorbent assay, *IL* interleukin, *MSD* meso scale discovery, *NLRP3* NOD-like receptor thermal protein domain associated protein 3, *PD* Parkinson’s disease, *PTX3* pentraxin 3, *SAA* serum amyloid A, *sAPPα* amyloid precursor protein-alpha, *sTREM2* soluble triggering receptor expressed on myeloid cells 2, *sVCAM-1* soluble vascular cell adhesion molecule-1, *S100β* central nervous system specific protein beta, *TNF* tumour necrosis factor, *YKL-40* chitinase protein 40.

### Diagnostic values of inflammatory biomarkers based on PD clinical features

We enroled studies that investigated inflammatory markers in relation to clinical features of motor and nonmotor symptoms, and a detailed overview is displayed in Tables 2–3. First, the systematic review summarized 36 records. Several studies have confirmed that abnormal IL-6, CRP, TNF-α, IL-4, IL-8, and TGF-β levels were associated with worse motor function assessed by the Unified Parkinson’s Disease Rating Scale (UPDRS), whereas CRP and fractalkine might be potential markers of freezing of gait (FOG). Research on nonmotor symptoms included 48 studies that focused on cognitive impairment, depression and anxiety, sleep disorders, fatigue, neuropsychiatric symptoms and autonomic function. Studies have reported that IL-6, TNF-α, CRP, YKL-40, IL-17, IL-1β, CCL2, IL-2, and IL‐8 are related to worse cognitive function or cognitive deterioration, while CRP, TNF-α, sIL-2R and CCL2 reflect severe symptoms of depression and anxiety. Sleep disorders, including RBD and ESS, exhibit significantly altered levels of IL-6, CRP, IL-1β, sTREM2, CCL3 and NO, suggesting these markers represent potential markers in PD patients. In addition, some inflammatory markers were closely associated with fatigue, hallucinations and illusions.

**Table 2:** The systematic review of the level changes for inflammatory markers on PD motor symptoms.

No.	Author	Year	Sample source	Assay type	Sample size	Age	Markers	Scale	Summary
**UPDRS scores**									
1	Mueller, T.	1998	CSF	ELISA	22	61 (1.5)	IL-6	UPDRS III	Significant inverse correlation.
2	Rentzos, M.	2007	Serum	ELISA	41	67.5 (8.1)	CCL5	UPDRS III	Strong and significant positive correlation.
3	Dufek, M.	2009	serum	CLIA	29	68.2 (5.5)	TNF-α	UPDRS III	No significant associations.
4	Rentzos, M.	2009	Serum	ELISA	41	65.8 (11.2)	IL-10, IL-12	UPDRS III	No significant associations.
5	Scalzo, P.	2010	Serum	ELISA	44	NA	IL-6	UPDRS III	No significant associations.
6	Hassin-Baer, S.	2011	Plasma	CLIA	73	68.8 (11.5)	CRP	UPDRS III	No significant associations.
7	Lee, H. W.	2011	Plasma	ELISA	66	65.8 (8.8)	PTX3	UPDRS III	Significant positive correlation.
8	Scalzo, P.	2011	Serum	ELISA	47	61.8 (10.7)	chemokines	UPDRS III	No significant associations.
9	Zhao, X. Q.	2012	Serum	ELISA	40	67.3 (9.4)	TNF-α, STNFR1, STNFR2	UPDRS III	No significant associations.
10	Tang, P.	2014	Serum	ELISA	78	76.3 (5.0)	CCL5	UPDRS III	No significant associations.
11	Jiang, Q. W.	2015	Plasma	ELISA	59	64.4 (8.1)	CCL3, CCL4	UPDRS III	No significant associations.
12	Martín de Pablos, A.	2015	CSF	ELISA	37	63.4 (0.9)	TGF-β1	UPDRS III	Positive correlation was found.
13	Umemura, A.	2015	Serum	NA	375	69.3	CRP	UPDRS III	Plasma CRP levels were associated with motor deterioration and predicted motor prognosis in patients with PD.
14	Hall, S.	2016	CSF	ELISA	63	64.7 (9.4)	YKL-40	UPDRS III	No significant associations.
15	Williams-Gray, C. H.	2016	Serum	V-PLEX	230	66.4 (9.5)	IFN-γ, IL, TNF-α, CRP	UPDRS III	IL-6 was associated with higher UPDRS-III motor scores, while TNF-α and CRP were correlated with faster rates of motor decline, and IL-13 with slower rate of motor decline.
16	Delgado-Alvarado, M.	2017	CSF/Plasma	Luminex Xmap	39	71.3 (6.2)	TNF-α, IL, IFN-γ	UPDRS III	Plasma IL-6 levels were positively correlated in PD patients with UPDRS III.
17	Kim, R.	2018	Serum	MSD	58	62.4 (8.1)	IL, TNF-α, CRP	UPDRS III	No significant associations.
18	Moghaddam, H. S.	2018	CSF	NA	109	69.7 (6.5)	CRP	UPDRS III	A significant correlation was observed.
19	Ahmadi Rastegar, D.	2019	Serum	Multiplex	65	NA	7 cytokines	UPDRS III	IL-5, IL-8, G-CSF, CCL2, IL-10, IFN-γ and IL-15 positively correlated with the fold change in UPDRS III.
20	Álvarez-Luquín, D. D.	2019	Plasma	ELISA	32	60.8 (10.2)	IL, IFN-γ, TNF-α, GM-CSF, TGF-β, IL-35	UPDRS III	The plasmatic levels of IL-17 positively correlated with the UPDRS III scores.
21	Green, H. F.	2019	Plasma	SIMOA	63	69.9 (8.1)	IL-6, IL-17A, TNF-α, TGF-β	UPDRS III	IL-6, TNF-α, IL-17A and TGF-β were correlated with UPDRS-III.
22	King, E.	2019	Serum	MSD	112	69.5 (6.7)	TNF‐α, IL, IFN‐γ, CRP	UPDRS III	Negative correlations between UPDRS III and IL‐2 and IL‐4.
23	Perner, C.	2019	Plasma	ELISA	33	69.6 (10.4)	sVCAM-1	UPDRS III	No significant associations.
24	Chatterjee, K.	2020	Serum	ELISA	27	62.5 (7.7)	IL-1β, NLRP3	UPDRS III	No significant associations.
25	Fan, Z	2020	Plasma	MSD	43	58.4 (1.4)	IL-1β	UPDRS III	A positive correlation was found between UPDRS III scores and plasma levels of IL-1β.
26	Peng, G.	2020	CSF/Plasma	ELISA	55	59.8 (8.9)	sTREM2	UPDRS III	No significant associations.
27	Santaella, A.	2020	CSF	ELISA	46	57.5 (10.0)	CCL2	UPDRS III	No significant associations.
28	Galper, J.	2021	Plasma	Bio-Plex	75	62.4 (1.2)	IL, TNF-α, chemokines, PDGF	UPDRS III	The UPDRS III score positively correlated to IL-4, IL-8, CCL2, TNF-α, and CCL3.
29	Li, S. Y.	2021	Serum	Nephelometry	148	63.8 (11.1)	CRP	UPDRS III	No significant associations.
30	Mo, M. S.	2021	CSF	ELISA	80	63.6 (8.5)	sTREM2	UPDRS III	No significant associations.
31	Zhu, Y.	2021	Serum	ELISA	46	69.5 (9.6)	IL-6, TNF-α, sLAG3	UPDRS III	TNF-α positively correlated with UPDRS III in PD patients.
32	Diaz, K.	2022	Serum	Milliplex	26	72.8 (7.1)	TNF-α, IFN-γ, IL, GM-CSF	UPDRS II&III	Higher levels of IL-4 and lower levels of IFN-γ significantly predicted more severe tremor in persons with PD.
33	Gupta, M.	2022	Serum	ELISA	21	57.9 (9.3)	CX3CL1	UPDRS III	Gradually falling CX3CL1 levels correlated with increasing motor aberrations in PD patients.
34	Imarisio, A.	2022	Plasma	Elecsys	71	65.1 (10.5)	IL-6, CRP	UPDRS III	IL-6 correlated with UPDRS-III.
35	Kaminska, M.	2022	serum	Multiplex	66	64.6 (9.8)	IL, TNF-α, BDNF	UPDRS III	IL-6 was associated with the UPDRS III.
36	Lerche, S.	2022	CSF	Multiplex	68	NA	ICAM-1, IL, CCL2, TNF-α	UPDRS III	Higher CSF levels of IL-8 and lower CSF levels of IL-18 were associated higher UPDRS-III scores.
**FOG**									
1	Santos-Garcia, D.	2019	Blood	ELISA	153	60.3 (6.1)	CRP	FOG-Q	CRP was significantly higher in PD patients with FOG, but it was not significant in the model after adjusting to covariates.
2	Hatcher-Martin, J. M.	2021	CSF	Milliplex	19	70.4 (10.1)	CX3CL1	NFOG-Q	CX3CL1 was significantly decreased in PD-FOG.
3	Liu, J.	2022	Plasma	Nephelometric	145	64.9 (11.0)	CRP	FOG-Q	The plasma CRP is a potential biomarker of FOG.

*BDNF* brain-derived neurotrophic factor, *CCL* chemokine (C-C motif) ligand, *CLIA* chemiluminescence immunoassay, *CRP* C-reactive protein, *CSF* cerebrospinal fluid, *CX3CL* CX3 chemokine ligand, *ELISA* enzyme-linked immunosorbent assay, *FOG* freezing of gait, *G-CSF* granulocyte colony-stimulating factor, *GM-CSF* granulocyte macrophage-colony stimulating factor, *IFN* interferon, *IL* interleukin, *MSD* Meso scale discovery, *NLRP3* NOD-like receptor thermal protein domain associated protein 3, *PD* Parkinson’s disease, *PDGF* platelet-derived growth factor, *PTX3* pentraxin 3, *UPDRS* Unified Parkinson’s Disease Rating Scale, *SIMOA* single molecular array, *sLAG3* soluble lymphocyte-activation gene 3, *STNFR* soluble tumour necrosis factor receptor, *sTREM2* soluble triggering receptor expressed on myeloid cells 2, *sVCAM-1* soluble vascular cell adhesion molecule-1, *TGF* transforming growth factor, *TNF* tumour necrosis factor, *YKL-40* chitinase protein 40.

**Table 3:** The systematic review of the level changes for inflammatory markers on PD non-motor symptoms.

No.	Author	Year	Sample source	Assay type	Sample size	Age	Markers	Scale	Summary
**Cognitive impairment**									
1	Selikhova, M. V.	2002	Plasma	ELISA	27	69.7 (8.9)	IL-6	MMSE	No significant associations.
2	Dufek, M.	2009	serum	CLIA	29	68.2 (5.4)	TNF-α	MMSE	No significant associations.
3	Menza, M.	2010	Plasma	ELISA	NA	NA	IL, TNF-α	MMSE	TNF-α was significantly correlated with cognition.
4	Scalzo, P.	2010	Serum	ELISA	44	NA	IL-6	MMSE	Higher levels of IL-6 were associated with poor cognitive function.
5	Hassin-Baer, S.	2011	Plasma	CLIA	73	68.8 (11.5)	CRP	MMSE	No significant associations.
6	Lee, H. W.	2011	Plasma	ELISA	66	65.8 (8.8)	PTX3	MMSE, CDR	No significant associations.
7	Lindqvist, D.	2013	CSF	MSD	71	64.1 (10.5)	CRP, IL, TNF-α, chemokines	MMSE	MMSE score correlated significantly with IL-6 levels.
8	Rocha, N. P.	2014	Plasma	ELISA	40	68.7 (10.1)	STNFR1, STNFR2	MMSE, FAB	STNFR1 was a significant predictor for FAB score.
9	Rocha, N. P.	2014	Plasma	ELISA	78	76.3 (5.0)	chemokines	MMSE	CXCL10 was associated with cognitive status.
10	Yu, S. Y.	2014	CSF	ELISA	26	57.4 (10.8)	IL, TNF-α, INF-γ	MoCA	Negative correlation between MoCA score and IL-6.
11	Jiang, Q. W.	2015	Plasma	ELISA	59	64.4 (8.1)	CCL3, CCL4	MMSE	No significant associations.
12	Park, S. J.	2015	Serum	NA	112	72.9 (5.7)	CRP	Diagnose	No significant associations.
13	Wennstrom, M.	2015	CSF	ELISA	61	68.4 (9.2)	YKL-40	MMSE	Negative correlation of CSF YKL-40 to MMSE.
14	Hall, S.	2016	CSF	ELISA	63	64.7 (9.4)	YKL-40	MMSE	An increase in YKL-40 correlated with worsening of cognitive function as measured by letter fluency.
15	Lue, L. F.	2016	Plasma	Multiplex	74	73.1 (1.3)	Cytokines, chemokines	Diagnose	A 14-protein panel with age served as discriminants of PD dementia.
								CDR	Significant associations of TNF-α, IL-2, CCL7, IL-17, CCL26, CCL13, IL-16 and BDNF.
								MMSE	Significant associations of IL-1β.
								AVLT-A7	Significant associations of CCL2, IL-17R, CCL11.
16	Williams-Gray, C. H.	2016	Serum	V-PLEX	230	66.4 (9.5)	IFN-γ, IL, TNF-α, CRP	MMSE	IFN-γ, TNF-α, IL-6, and CRP levels were associated with lower MMSE scores, while IL-1β and IL-2 were correlated with faster rate of cognitive decline.
17	Hall, S.	2018	CSF	MSD	131	64.9 (10.6)	CRP, SAA, YKL-40, CCL2	Diagnose	CRP and SAA were higher in patients with PD dementia. The levels of CCL2 in CSF were lower in PD dementia.
18	Karpenko, M. N.	2018	Serum	ELISA	117	65 (57-73)	IL, TNF-α	MMSE	The serum level of TNF-α was significantly lower in PD patients with MCI.
19	Kim, R.	2018	Serum	MSD	58	62.4 (8.1)	IL, TNF-α, CRP	MoCA	No significant associations.
20	Moghaddam, H. S.	2018	CSF	NA	109	69.7 (6.5)	CRP	MoCA	A significant correlation was observed.
21	Rocha, N. P.	2018	Plasma	CBA	40	68.7 (10.1)	IL, TNF, IFN-γ	MMSE	Higher TNF/IL-10 ratios were associated with worse cognitive performance.
22	Veselý, B.	2018	Serum	CLIA	47	65 (7.8)	IL-6	MMSE	No significant associations.
23	Green, H. F.	2019	Plasma	SIMOA	63	69.9 (8.1)	IL, TNF-α, TGF-β	MoCA	IL-17A was negatively correlated with MoCA score.
24	King, E.	2019	Serum	MSD	112	69.5 (6.7)	TNF‐α, IL, IFN‐γ, CRP	MoCA	IL‐8 was significantly higher in PD without MCI.
25	Chatterjee, K.	2020	Serum	ELISA	27	62.5 (7.7)	IL-1β, NLRP3	MMSE, DRS-2	No significant associations.
26	Kiçik, A.	2020	Serum	ELISA	61	NA	NLRP3, IL-1β, IL-18	Diagnose	PD-MCI patients displayed significantly reduced serum IL-1β and IL-18 levels.
27	Martin-Ruiz, C.	2020	Serum	MSD	154	67 (60-82)	CRP, IL-6	MMSE, MoCA	Levels of CRP and IL-6 were significantly raised in PD-MCI cases.
28	Santaella, A.	2020	CSF	ELISA	46	57.5 (10.0)	CCL2	MMSE	No significant associations.
29	Bartl, M.	2021	CSF	ELISA	252	61 (9.8)	GFAP, S100β, YKL-40, sTREM2	MoCA	The MoCA score showed a significant negative correlation with GFAP, S100, YKL-40 and sTREM2.
30	Galper, J.	2021	Plasma	Bio-Plex	75	62.4 (1.2)	TNF-α, IL, chemokines, PDGF	MoCA	MoCA score significantly negatively correlated to IL-17RA, CXCL10, CCL3, and CCL18, and positively correlated to PDGF.
31	Lerche, S.	2022	CSF	Multiplex	68	-	ICAM-1, IL, CCL2, TNF-α	MoCA	Higher CSF levels of IL-8 and CCL2 were associated with lower MoCA scores.
32	Li, Y. Y.	2022	Plasma	MSD	76	62.2 (7.5)	Chemokines, IL-8	MMSE	An increase in CCL15 levels was associated with an increased MMSE score.
**Depression and anxiety**									
1	Selikhova, M. V.	2002	Plasma	ELISA	27	69.7 (8.9)	IL-6	BDI	Significant positive association.
								STAI	No significant associations.
2	Menza, M.	2010	Plasma	ELISA	NA	NA	IL, TNF-α	HAMD	TNF-α was significantly correlated with depression.
3	Pålhagen, S.	2010	CSF	EIAs	25	64.9 (8.4)	IL-6	HAMD MADRS	No significant associations.
4	Lindqvist, D.	2012	Serum	MSD	86	64.2 (10.8)	CRP, IL-6, IL-2R, TNF-α	HADS	TNF-α and sIL-2R were positively correlated with HAD depression scores.
									TNF-α and sIL-2R were positively correlated with HAD anxiety scores.
5	Lindqvist, D.	2013	CSF	MSD	71	64.1 (10.5)	CRP, IL-6, TNF-α, chemokines	HADS	HADS depression score correlated positively with CRP and MCP-1 and IP-10.
6	Rocha, N. P.	2014	Plasma	ELISA	78	76.3 (5.0)	chemokines	BDI	No significant associations.
7	Jiang, Q. W.	2015	Plasma	ELISA	59	64.4 (8.1)	CCL3, CCL4	HAMD	MIP-1α was correlated with depression in early PD.
8	Li, Z. J.	2016	Serum	ELISA	65	64.6 (8.2)	IL-6, IL-18, TNF-α, CRP	HAMD	Serum IL-6, IL-1β, TNF-α and CRP were significantly higher.
9	Wang, X. M.	2016	Blood	ELISA	62	65.0 (7.2)	IL-1β, IL-6, INF-γ, CRP, sIL-2R	HAMD	HAMD scores were positively correlated with the levels of TNF-α, CRP and sIL-2R of PD patients.
								HAMA	HAMA scores were positively correlated with the levels of TNF-α, CRP and sIL-2R of PD patients.
10	Hall, S.	2018	CSF	MSD	131	64.9 (10.6)	CRP, SAA, YKL-40, CCL2	HADS	Increased depressive symptoms correlated with CRP and SAA.
11	Karpenko, M. N.	2018	Serum	ELISA	117	65 (57-73)	IL, TNF-α	HADS	A direct correlation was only found between the level of serum IL-10 and depression.
								HADS	A correlation was found between the level of serum IL-10 and anxiety.
12	Veselý, B.	2018	Serum	CLIA	47	65 (7.8)	IL-6	MADRS	Patients with higher IL-6 at baseline showed worse depression scores at 2 years.
13	Ahmadi Rastegar, D.	2019	Serum	Multiplex	65	NA	IL, G-CSF, chemokines, TNF-α, FGF basic, VEGF	GDS	Fourteen cytokines positively correlated with the fold change in geriatric depression scale over the 2-year time period.
14	Green, H. F.	2019	Plasma	SIMOA	63	69.9 (8.1)	IL, TNF-α, TGF-β	HADS	No significant associations.
								HADS	IL-17A was positively correlated with the anxiety subscale of HADS.
15	Lian, T. H.	2020	CSF	ELISA	86	62.2 (9.5)	TNF-α	HAMD	TNF-α played an important role in PD depression.
16	Zhu, Y.	2021	Serum	ELISA	46	69.5 (9.6)	IL-6, TNF-α, sLAG3	HAMD	No significant associations.
								HAMA	Serum TNF-α and sLAG3 positively correlated with HAMA.
**Sleep disorders**									
1	Menza, M.	2010	Plasma	ELISA	NA	NA	IL, TNF-α	PSQI	No significant associations.
2	Hassin-Baer, S.	2011	Plasma	Chemical	73	68.8 (11.5)	CRP	Self-reported	No significant associations.
3	Lindqvist, D.	2012	Serum	MSD	86	64.2 (10.8)	CRP, IL, TNF-α	SCOPA-S	No significant associations.
4	Hu, Y.	2015	CSF	ELISA	84	NA	NO, H_2_O_2_, IL-1β, TNF-α	RBDSQ	Enhanced RBDSQ scores with elevated levels of NO and IL-1β in the CSF of patients with PD.
5	Jiang, Q. W.	2015	Plasma	ELISA	59	64.4 (8.1)	CCL3, CCL4	RBDSQ	CCL3 was correlated with RBD in early PD.
6	Hu, Y.	2021	CSF/Serum	ELISA	139	NA	IL-1β, TNF-α	EDS	ESS scored higher as IL-1β concentration in CSF elevated in patients with PD.
7	Mo, M. S.	2021	CSF	ELISA	80	63.6 (8.5)	sTREM2	PDSS	PD patients with a moderate or severe sleep disorder had a significantly increased concentration of sTREM2 in their CSF.
8	Kaminska, M.	2022	Serum	Multiplex	66	64.6 (9.8)	IL, TNF-α, BDNF	Polysomnography	IL-6 was associated with some polysomnographic characteristics.
								ESS	No significant associations.
9	Wang, L. X.	2022	Blood	NA	93	61 (51-68)	CRP	NA	CRP levels served as biomarkers and predicted the prognosis of PD patients with RBD.
10	Yuan, Y.	2022	CSF/Serum	EIA	13	NA	TNF-α	RBDQ	No significant associations.
**Fatigue**									
1	Lindqvist, D.	2012	Serum	MSD	86	64.2 (10.8)	CRP, IL-6, TNF-α, chemokines	FACIT	TNF-α and sIL-2R were positively correlated with FACIT scores.
2	Lindqvist, D.	2013	CSF	MSD	71	64.1 (10.5)	CRP, IL-6, TNF-α, chemokines	FACIT	FACIT score correlated negatively with CRP, CXCL10, and CCL2.
3	Pereira, J. R.	2016	Serum	ELISA	44	65.1 (10.9)	IL-6, STNFR1, STNFR2	PFS	Fatigued PD patients have elevated IL-6 serum levels when compared with non-fatigued patients.
4	Hall, S.	2018	CSF	MSD	131	64.9 (10.6)	CRP, SAA, YKL-40, CCL2	FACIT	Increased fatigue symptoms correlated with CRP and SAA.
**Neuropsychiatric symptoms**									
1	Hassin-Baer, S.	2011	Plasma	Chemical	73	68.8 (11.5)	CRP	PPRS, AS, BDI	No significant associations.
2	Sawada, H.	2014	Plasma	-	111	69.7 (7.8)	CRP	PPQ-A	Subclinical elevations of CRP levels might be an independent risk for hallucinations/illusions.
3	Wang, Y. H.	2015	Plasma	Nephelometric	62	65.8 (9.3)	CRP, IL-6	PPQ-B	The levels of IL-6 and CRP were significantly higher in hallucination group.
**Autonomic function**									
1	Jiang, Q. W.	2015	Plasma	ELISA	59	64.4 (8.1)	CCL3, CCL4	SCOPA-AUT	No significant associations.

*AS* apathy scale, *AVLT* auditory verbal learning test, *BDI* Beck depression inventory, B*DNF* brain-derived neurotrophic factor, *CBA* cell based assay, *CCL* chemokine (C-C motif) ligand, *CDR* clinical dementia rating, *CLIA* chemiluminescence immunoassay, *CRP* C-reactive protein, *CSF* cerebrospinal fluid, *CX3CL* CX3 chemokine ligand, *EDS* excessive daytime sleepiness, *ELISA* enzyme-linked immunosorbent assay, *ESS* Epworth sleepiness scale, *FAB* frontal assessment battery, *FACIT* the functional assessment of chronic illness therapy-fatigue, *FGF-basic* fibroblast growth factor-basic, *FOG* freezing of gait, *G-CSF* granulocyte colony-stimulating factor, *GFAP* glial fibrillary acidic protein, *GM-CSF* granulocyte macrophage-colony stimulating factor, *HADS* hospital anxiety and depression scale, *HAMA* Hamilton anxiety scale, *HAMD* Hamilton depression scale, *IFN* interferon, *IL* interleukin, *MARDS* Montgomery-Asberg depression rating scale, *MCI* mild cognitive impairment, *MMSE* mini-mental state examination, *MoCA* Montreal cognitive assessment, *MSD* Meso scale discovery, *NLRP3* NOD-like receptor thermal protein domain associated protein 3, *PD* Parkinson’s disease, *PDSS* Parkinson’s disease sleep scale, *PDGF* platelet-derived growth factor, *PPQ* Parkinson psychosis questionnaire, *PRRS* Parkinson psychosis rating scale, *PSP* Piper fatigue scale, *PSQI* Pittsburgh sleep quality index, *PTX3* pentraxin 3, *RBDSQ* REM Sleep behavior disorder screening questionnaire, *UPDRS* Unified Parkinson’s Disease Rating Scale, *SAA* serum amyloid A, *sAPPα* amyloid precursor protein-alpha, *SCOPA-AUT* scales for outcomes in Parkinson’s disease-autonomic, *SCOPA-S* scales for outcomes in Parkinson’s disease-sleep, *SIMOA* single molecular array, *sLAG3* soluble lymphocyte-activation gene 3, *STAI* state-trait anxiety inventory, *STNFR* soluble tumour necrosis factor receptor, *sTREM2* soluble triggering receptor expressed on myeloid cells 2, *sVCAM-1* soluble vascular cell adhesion molecule-1, *S100β* central nervous system specific protein beta, *TGF* transforming growth factor, *TNF* tumour necrosis factor, *VEGF* vascular endothelial growth factor, *YKL-40* chitinase protein 40.

### Functional enrichment and protein‒protein interaction (PPI) network construction analyses

**Fig. 5:**
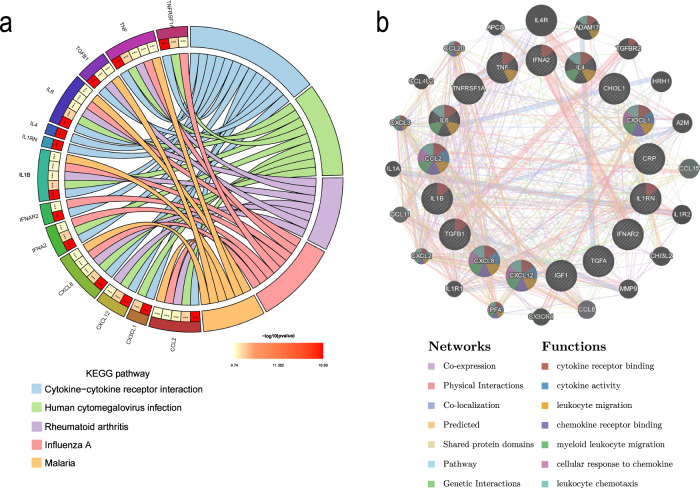
The KEGG pathway enrichment analysis and PPI network construction analysis of inflammatory markers. The KEGG pathway enrichment analysis showed that the inflammatory markers related to PD were mainly involved in cytokine–cytokine receptor interactions, human cytomegalovirus infection, rheumatoid arthritis, influenza A and the malaria pathway (**a**). The PPI analysis revealed that the major functions were involved in cytokine receptor binding, cytokine activity, leucocyte migration, chemokine receptor binding, myeloid leucocyte migration, cellular response to chemokine and leucocyte chemotaxis (**b**).

Based on the identified proteins, we conducted Kyoto Encyclopedia of Genes and Genomes (KEGG) pathway enrichment analyses to predict the potential function of robust markers. KEGG pathways with adjusted *P* < 0.05 were considered statistically significant. The results of KEGG pathway enrichment analysis showed that these markers were mainly involved in cytokine‒cytokine receptor interactions, human cytomegalovirus infection, rheumatoid arthritis, influenza A and the malaria pathway (Fig. 5a).

PPI network analysis was performed using the Search Tool for the Retrieval of Interacting Genes (STRING) to predict protein functional associations. The interaction network of overlapping targets with a combined score of >0.4 was considered statistically significant. Subsequently, the network was imported into Cytoscape software for visualization^11^. As shown in Fig. 5b, the network contains 37 nodes and 396 edges. The analysis revealed that the following functions were involved based on the 17 most significant targets: cytokine receptor binding, cytokine activity, leucocyte migration, chemokine receptor binding, myeloid leucocyte migration, cellular response to chemokine and leucocyte chemotaxis. Furthermore, the results of 39 potential markers are shown in Supplementary Fig. 2.

## Discussion

Our meta-analysis comprehensively demonstrated multiple significant differences in inflammatory biomarker levels in peripheral blood and CSF between the PD and control groups. As noted, several potential markers were identified based on their ability to differentiate PD patients from healthy controls with good performance. Moreover, some of these inflammatory markers might represent biomarkers of clinical features, including motor and nonmotor symptoms. These findings suggested noteworthy blood and CSF alterations in inflammatory markers in PD patients, implying the important role of inflammation in PD pathology, and providing optimal biomarkers for the early disease diagnosis and monitoring.

To the best of our knowledge, this meta-analysis performed the most comprehensive evaluation to investigate the changes in peripheral inflammatory markers of PD. We found significant increases in inflammatory cytokine levels (IL-6, IL-1β and TNF-α) in both peripheral blood and CSF among patients with PD compared to healthy controls. These findings are consistent with previous meta-analyses^9,10^. Levels of the chemokine CCL2, also named monocyte chemoattractant protein-1 (MCP-1), which is associated with the recruitment of monocytes and T cells to sites of inflammation, were also increased in PD patients. However, inconsistent results have been reported in which only a few inflammatory markers showed significant differences in blood or CSF. We also found increased blood chemokine concentrations of CX3CL1 (fractalkine) and CXCL12 (stromal-derived factor [SDF]-1) as well as reduced cytokine levels of IL-4 and IFN-γ, in the PD group compared with the control group. In addition, IL-2 and CCL5 (RANTES) levels were previously reported to be elevated in patients with PD^9^, but significant differences were not observed in our study. This finding was likely attributed to the larger sample size and stricter inclusion criteria of this analysis. The increased levels of CRP in blood and CSF were verified in our study and a previous meta-analysis^12^, strengthening the clinical evidence that patients with PD exhibit increased inflammatory activation.

It has been verified that the expression and peripheral levels of proinflammatory cytokines and chemokines are significantly increased in patients with PD, which have been broadly documented to correlate with the hypothesis that α-synuclein in the brain directly activates microglia^13^. However, clinical alterations in these markers and their effects on PD progression are controversial. First, cytokines promote the apoptosis of neurons, oligodendrocytes and astrocytes, damage myelinated axons; and even initiate neuroprotective effects. These effects occur independent of the immunoregulatory properties of cytokines^14^. The most studied cytokines in PD are IL-6, IL-1β and TNF-α, and the role of IL-6 is distinct from that of IL-1β and TNF-α based on the contributions of its pro-and anti-inflammatory functions to neuropathology^15,16^. A previous study observed an increase in IL-6 in the SN region of the postmortem brain of PD patients^17^, and IL-6 plasma levels are also related to PD progression^18^. Similarly, a model of neuron cultures shows that chronic exposure to IL-6 during neuronal development can lead to cell damage and death in a subpopulation of developing granule neurons^19^. This meta-analysis discovered elevated peripheral levels of IL-6 in patients with PD. Thus, we suggest that enhanced circulating levels of IL-6 may be proinflammatory, leading to the progression of PD pathophysiology. On the other hand, the major proinflammatory factors IL-1β and TNF-α can induce oxidative stress, neuronal death and in particular the loss of dopaminergic neurons in PD^20,21^. It has been reported that sustained expression of IL-1β in the SN causes irreversible and pronounced dopaminergic neuronal loss and motor symptoms of PD^22^. Furthermore, treatments that reduce IL-1β and TNF-α levels may significantly improve motor function in PD mice^23^. TNF-α and its receptor sTNFR1, which regulate numerous physiological processes in the CNS, exacerbate the main pathological changes of PD (progressive loss of dopaminergic neurons) in vivo^24^. Our meta-analyses demonstrated notable differences in the peripheral concentrations of cytokines, implying that these markers might be useful in monitoring disease deterioration.

Second, only a few studies have evaluated circulating levels of chemokines in PD patients, and the results are inconclusive. Interestingly, elevated MCP-1 levels were found in the peripheral blood and CSF of patients with PD compared with controls according to our findings. MCP-1, one of the most highly and transiently expressed chemokines during inflammation, has been implicated in many neurodegenerative disorders through the regulation of monocyte chemotaxis and endothelial activation^25^. Preclinical studies in mouse models suggest that MCP-1 causes neuronal leakage through the blood-brain barrier (BBB) and macrophage polarization^26^ and promotes the continuous differentiation of dopamine precursors and neurogenesis of dopaminergic neurons in the midbrain^27^. Additionally, a clinical study illustrated a positive association between MCP-1 and nonmotor symptoms^28^. Other chemokines, such as fractalkine and SDF-1, are increased in the peripheral blood of PD subjects. Emerging evidence suggests the crucial role of fractalkine in neuron-to-glia communication signalling in PD^29^, and SDF-1 is correlated with the apoptosis of PD-related neurons by activating chemokine receptor 4 (CXCR4)^30^.In contrast to our findings, previous studies have reported that peripheral RANTES is significantly elevated and suggested that CCL5 produced from the CNS penetrates into the serum through the BBB^31^. These inflammatory targets provide further opportunities to explore their promising therapeutic values in PD.

Anti-inflammatory strategies are also considered beneficial for PD. Our results revealed controversial findings for the anti-inflammatory marker IL-4 in peripheral blood and CSF, possibly suggesting dual functions in the CNS. IL-4 shapes microglial functions to promotes the survival of dopaminergic neurons in animal models^32^, which underlines the therapeutic potential of IL-4 administration in PD. In addition, IL-4 promotes neurodegeneration in proinflammatory rat models by contributing to microglial activation, IL-1β production, and BBB disruption^33^. In addition, the peripheral levels of IFN-γ unexpectedly exhibited diverse alterations. Past studies reported that IFN-γ deficiency attenuated dopaminergic lesions in PD models by inhibiting microgliosis and inducible NO synthase (iNOS) expression, indicating that IFN-γ may contribute to dopaminergic loss by acting through microglial activation^34,35^. However, IFN-γ increases the proliferation of neural precursor cells and enhances neurogenesis in AD models^36^. Current studies do not entirely disclose how peripheral markers of inflammation reflect neuroinflammation activity. Hence, the inconsistent results for these markers in the CNS and peripheral blood system urgently need to be explored in future studies.

Most of the studies have consistently demonstrated obviously increased CRP levels both in blood and CSF in patients with PD. Some scholars hold the view that CRP can also be generated by neurons and microglia in the CNS^37^, and epidemiological studies observe that long-term anti-inflammatory medication therapy is beneficial and will delay or prevent dopaminergic cell death by inhibiting the proinflammatory responses of microglia^38^. However, others believe that patients with PD are more susceptible and have a higher infectious burden than health individuals^39^. Taken together, the present analyses cannot completely determine the actual mechanisms of these proteins in PD initiation and progression.

The network construction assists us better understand the interaction among inflammatory markers and aim at fresh therapeutic targets of PD. For instance, the NF-κB pathway participates in microglia activation and consequently gives rise to the release of multiple pro-inflammatory and anti-inflammatory cytokines^40^, and can subsequently release chemokines and recruit peripheral immune cells, indicating the joint effort of cytokines and chemokines of inflammation in PD. The inflammatory markers also take part in other immune reaction like leucocyte migration and leucocyte chemotaxis^41^, which reflects the diverse function of them.

Given the variety of studies included in this meta-analysis, it is inevitable that each cytokine will exhibit heterogeneity. However, techniques are currently being developed achieve greater sensitivity, and ultrasensitive platforms, including Luminex XMAP, Meso Scale Discovery (MSD) and Simoa (Single Molecular Array), have appeared. These platforms facilitate the detection of multiple markers in the same sample and overcome issues associated with low levels of target biomarkers. Here, we conducted subgroup analyses based on detection techniques to adjust for potential confounders. However, the results were not consistent with our expected findings for the combined data for inflammatory markers measured by multiplex assays, as obvious heterogeneity remained. We hypothesize that these discrepancies are partly attributed to the sensitivities of the various assays used and patient characteristics.

Inflammation can also reflect more advanced motor and nonmotor symptom processes. We conclude that a number of inflammatory markers in blood and CSF are associated with more severe motor and nonmotor symptoms, whereas some are able to predict symptomatic progression. Exploring the diagnostic and prognostic values of inflammatory markers for clinical symptoms is essential but still inadequate; therefore, future research may pay more attention to the clinical features of PD to enrich maximize the therapeutic benefit. In addition, the combined diagnosis is augmented largely by the use of multiple cytokines and chemokines, such as α-synuclein and AD core biomarkers, as well as the type variances. These results imply that multiplex assays measuring various inflammatory markers can serve as appropriate detection approaches.

Limitations to our meta-analysis should be noted. The foremost weakness is the lack of relative studies for some newly identified markers. Due to the limited availability of information, this study is underpowered to investigate alterations in these inflammatory markers in PD. Thus, future studies should better address these aspects. Next, large differences were noted based on measurement approaches, so multiplex assays should be validated in larger cohorts and more unified operating platforms should be employed. Finally, certain eligible articles and inflammatory markers might be missed even though systemic research was performed, and a portion of the articles identified reported results in the form that was inappropriate for the present meta-analysis, which would potentially bias our results.

In summary, our meta-analysis demonstrated altered IL-6, TNF-α, IL-1β, MCP-1 and CRP levels in both peripheral blood and CSF in PD patients versus control groups, and altered IL-4, IFN-γ, STNFR1 and fractalkine only in blood. These findings based on a large sample size strengthen the clinical evidence that PD is accompanied by a specific peripheral inflammatory response.

## Methods

### Search strategy and selection criteria

This systematic review and meta-analysis was performed according to the Preferred Reporting Items for Systematic Reviews and Meta-Analysis (PRISMA) 2009 guidelines (Supplementary Table 6)^42^. Electronic databases (PubMed, Cochrane Library, Embase and Web of Science) were systematically searched for studies that reported data of inflammatory biomarkers in peripheral blood and CSF for patients with PD versus controls from database inception to June 8, 2022. The initial study protocol was preregistered at PROSPERO (CRD42022349182). The full search strategy is listed in Supplementary Table 7, and additional literature was added by hand-searching references of relevant reviews and meta-analyses.

Studies were included if they met the following criteria: (a) original studies reported data about concentrations of inflammatory markers in at least two of the groups (PD and control); (b) literature sources and necessary data were met; and (c) the principles of PD diagnosis were qualified. Studies were excluded for the following reasons: (a) measured marker concentrations in postmortem samples, animals or in vitro; (b) duplicated samples that overlapped with other studies; and (c) raw data could not be obtained completely. For several publications reported from the same centre, we included the publication that had greatest sample size.

### Data extraction

Data including study characteristics (i.e., first author, publication year, study design, sample size, age, sex and region), information for potential moderator analysis (i.e., sample sources and assay types) and PD assessments (i.e., diagnostic criteria, disease duration, Hoehn-Yahn stages and UPDRS III scores), were independently extracted by two researchers. Biomarkers are presented as concentrations with the mean (SD [standard deviation]), median (IQR [interquartile range]) or median (range), and the data of the latter is converted to the former by using a new evaluative method^43^. All data and any controversies were checked and resolved by a third author.

### Quality assessment of studies

The Newcastle‒Ottawa Scale (NOS) was used for quality assessments of all potentially eligible studies^44^. The scale ranges from 0 to 9 stars and awards four stars for selection of study participants, two stars for comparability of studies, and three stars for the adequate ascertainment of outcomes. Studies with NOS scores <6 were recognized to be of low quality and therefore excluded.

### Statistical analysis

All statistical analyses were conducted using Comprehensive Meta-Analysis Software (version 3) and GraphPad Prism (version 8). Effect sizes (ESs) were primarily adopted from sample sizes and mean (SD) values of cytokine concentrations between patients with PD and controls. Additionally, ESs were calculated from sample size and *P*-values if mean (SD) data were not available. Hedges’ g values were performed as the combined ESs to reduce the potential biases^45^, and random effects meta-analysis was used in all analyses. Heterogeneities among studies were assessed using the Cochrane Q test and *I*^2^ index. *P* < 0.10 indicated a significant difference for the Cochrane Q, and *I*^2^ index values 0.25, 0.50, and 0.75 distinguished small, moderate, and high levels of heterogeneity, respectively. Publication bias was conducted to assess if whether the pooled effect values were impacted by parts of the studies’ positive results and assessed by Egger’s test (>3 studies). Then, subgroup analysis was employed to significantly reduce the heterogeneity and publication bias within every subgroup. In addition, inflammatory markers measured in one study were assessed qualitatively in the systematic review. *P*-values of 0.05 or less were considered significant.

### Reporting summary

Further information on research design is available in the Nature Research Reporting Summary linked to this article.

## Supplementary information

Reporting Summary

Supplementary

## Data Availability

All data generated or analysed during this study are included in this published article (and its supplementary information files).
