# Supplementary material for: A systematic review and meta-analysis of inflammatory biomarkers in Parkinson’s disease
Source: NPJ Parkinsons Dis. 2023 Feb 4;9:18. doi: 10.1038/s41531-023-00449-5 (PMC9899271; doi:10.1038/s41531-023-00449-5)
Supplement: Supplementary file 2 — Supplementary [file 41531_2023_449_MOESM2_ESM.pdf]

Title: Fluid Biomarkers of Inflammation in Parkinson's Disease: A Systematic Review and Meta-Analysis

CONTENT

TITLE	PAGE
Supplementary Table 1. Characteristics of included studies.	2 to 23
Supplementary Table 2. Quality assessment.	24 to 27
Supplementary Table 3. Official cytokine names.	28 to 30
Supplementary Table 4. Summary of comparative outcomes of peripheral blood cytokines levels in individuals with PD versus controls.	31 to 33
Supplementary Table 5. Summary of comparative outcomes of cerebrospinal fluid cytokines levels in individuals with PD versus controls.	34 to 35
Supplementary Table 6. PRISMA 2009 Checklist.	36 to 38
Supplementary Table 7. Search strategy.	39
Supplementary Figure 1. Comparative outcomes of peripheral blood cytokines levels for PD patients versus controls (not significant).	40
Supplementary Figure 2. Comparative outcomes of peripheral blood cytokines levels for PD patients versus controls in the systematic review (not significant).	41
Supplementary Figure 3. Comparative outcomes of cerebrospinal fluid cytokines levels for PD patients versus controls (not significant).	43
Supplementary Figure 4. Comparative outcomes of cerebrospinal fluid cytokines levels for PD patients versus controls in the systematic review (not significant).	43
Supplementary Figure 5. KEGG pathway enrichment analysis and PPI network construction analysis.	44

Supplementary Table 1. Characteristics of included studies.

No.	Author	Year	Region	Sample source	Assay Type	Samples, n		Age, y		Gender		PD evaluations				Ref.
						PD	HC	PD	HC	PD	HC	Criteria	Duration, y	H-Y	UPDRS-III	
1	Mogi, M.	1994	Japan	CSF	ELISA	15	16	58 (40-70)	46 (25-75)	M6 F9	M6 F10	NA	1-30	NA	NA	[1]
2	BlumDegen, D.	1995	Germany	CSF	ELISA	22	12	61 (1.5)	61 (4.2)	M12 F10	M6 F6	NA	0.5-3	NA	NA	[2]
3	Klueter, H.	1995	Germany	Blood	ELISA	20	19	63.3 (5.0)	61.1 (3.2)	M11 F9	M12 F8	UKPDSBBC	6.7 (1-17)	1-4	NA	[3]
4	Mogi, M.	1996	Japan	VCSF	ELISA	14	13	68 (54-75)	46 (14-65)	M7 F7	M10 F3	NA	NA	NA	NA	[4]
5	Stypula, G.	1996	Poland	Plasma	RIA	21	13	64.6 (2.0)	46 (14-65)	M14 F7	M10 F3	NA	NA	1-3	NA	[5]
6	Vawter, M. P.	1996	USA	CSF	ELISA	30	16	73.8 (1.4)	72.7 (3.1)	M22 F8	M11 F5	NA	NA	NA	NA	[6]
7	Mueller, T.	1998	Germany	CSF	ELISA	22	22	61 (1.5)	NA	NA	NA	NA	NA	NA	NA	[7]
8	Dobbs, R. J.	1999	Britain	Serum	ELISA	78	140	69.4 (12.3)	61 (16.6)	M43 F35	M68 F72	NA	NA	2-4	NA	[8]
9	Wandinger, K. P.	1999	Germany	Blood	ELISA	10	16	66.8 (11.8)	61.2 (9.6)	M6 F4	NA	UKPDSBBC	1.9 (1.5)	1-3	NA	[9]
10	Yang, Z. Z.	2001	China	CSF/ Serum	ELISA	18	16	59 (46-76)	33 (18-46)	M12 F6	M9 F7	Chinese criteria	NA	NA	NA	[10]
11	Selikhova, M. V.	2002	Russia	Plasma	ELISA	27	15	69.7 (8.9)	62.5 (8.6)	NA	NA	NA	NA	40.2 (18.3)	NA	[11]
12	Rota, E.	2006	Italy	CSF/	RIA	14	25	66 (6.4)	69 (8.6)	M9	M15	NA	3.0 (2.1)	NA	NA	[12]

				Serum						F5	F10					
13	Rentzos, M.	2007	Greece	Serum	ELISA	41	19	67.5 (8.1)	65.8 (11.2)	M20<br>F21	M9<br>F10	Larsen JP's criteria	4 (1.5-7)	2.5 (1–4)	25 (18-32)	[13]
14	Brodacki, B.	2008	Poland	Serum	CBA	55	20	NA	NA	NA	NA	UKPDSBBC	NA	2.6 (0.2)	42 (18)	[14]
15	Chen, H. L.	2008	USA	Plasma	NA	84	165	67.2 (7.6)	67.2 (7.5)	NA	NA	Medical records	NA	NA	NA	[15]
16	Choi, C.	2008	Korea	CSF/<br>Serum	Luminex Xmap	8	13	73.0 (8.6)	68.5 (7.2)	M0<br>F8	M5<br>F8	UKPDSBBC	NA	NA	NA	[16]
17	Xu. J. P.	2008	China	Serum	RIA	51	35	67 (12.1)	57.7 (11.1)	M29<br>F22	NA	Chinese criteria	NA	NA	NA	[17]
18	Dufek, M.	2009	Czech Republic	Serum	EIAs	29	-	68.2 (5.4)	-	M21<br>F8	-	UKPDSBBC	9.3 (4.4)	3 (0.6)	19 (12.2)	[18]
19	Hofmann, K. W.	2009	Brazil	Serum	ELISA	40	23	66.0 (10.7)	60.0 (7.8)	M22<br>F18	M15<br>F8	UKPDSBBC	5.8 (5.9)	NA	NA	[19]
20	Rentzos, M.	2009	Greece	Serum	ELISA	41	19	67.5 (8.1)	65.8 (11.2)	M20<br>F21	M9<br>F10	Larsen JP's criteria	4 (1.5-7)	2.5 (1-4)	25 (18-32)	[20]
21	Scalzo, P.	2009	Brazil	Serum	ELISA	46	23	61.9 (10.8)	65.8 (8.9)	M23<br>F23	M8<br>F15	NA	7.5 (4.6)	2 (1-4)	34.9 (22.4)	[21]
22	Tuncel, D.	2009	Turkey	Serum	Chemical	212	119	68.7 (9.3)	66.6 (12.6)	M77<br>F135	M59<br>F60	UKPDSBBC	2.1 (1.1)	NA	NA	[22]
23	Menza, M.	2010	USA	Plasma	ELISA	52	-	62.8	-	M25<br>F27	-	UKPDSBBC	6.6	2.2	NA	[23]
24	Pålhagen, S.	2010	Sweden	CSF	EIAs	25	-	64.9 (8.4)	-	M14<br>F11	-	UKPDSBBC	8.1 (3.8)	2.0 (0.4)	23.6 (10.3)	[24]
25	Scalzo, P.	2010	Brazil	Serum	ELISA	44	22	NA	NA	NA	NA	NA	NA	NA	NA	[25]
26	Varani, K.	2010	Italy	Serum	ELISA	56	60	74.2 (8.1)	71.4 (8.3)	M38	M40	NA	NA	NA	NA	[26]

									F18	F20						
27	Hassin-Baer, S.	2011	Israel	Plasma	Chemical	73	-	68.8 (11.5)	-	M46 F27	-	UKPDSBBC	6.7 (4.7)	2 (2–3)	24.2 (12.3)	[27]
28	Lee, H. W.	2011	Korea	Plasma	ELISA	66	41	65.8 (8.8)	64.7 (6 7)	M26 F40	M15 F26	UKPDSBBC	NA	NA	NA	[28]
29	Scalzo, P.	2011	Brazil	Serum	ELISA	47	23	61.8 (10.7)	65.7 (8.8)	M24 F23	M8 F15	NA	7.6 (4.5)	2 (1-4)	34.5 (22.3)	[29]
30	Shi, M.	2011	USA	CSF	Luminex Xmap	126	137	63.8 (10.4)	58.9 (18.4)	M88 F29	M52 F53	UKPDSBBC	NA	NA	NA	[30]
31	Andican, G.	2012	Turkey	Plasma	ELISA	45	25	63.6 (13.5)	60.2 (12.5)	M25 F20	M15 F10	NA	6.4 (4.3)	6.4 (4.3)	NA	[31]
32	Gruden, M. A.	2012	Russia	Serum	ELISA	32	26	60.8 (2.0)	63.0 (3.0)	M20 F12	M19 F7	NA	1-5	2.1 (0.6)	23.3 (1.9)	[32]
33	Koziorowski, D.	2012	Poland	Serum	Luminex Xmap	60	24	59 (15.5)	64 (5.8)	M30 F30	M12 F12	UKPDSBBC	11.3 (10.7)	2.4 (0.8)	NA	[33]
34	Lindqvist, D.	2012	Sweden	Serum	MSD	86	40	64.2 (10.8)	64.8 (9.0)	M51 F35	M14 F26	NA	6.9 (6.2)	1.9 (0.9)	17.3 (10.4)	[34]
35	Liu, J.	2012	China	Serum	Nephelometric	20	20	62 (5.6)	60.2 (8.2)	M12 F8	M13 F7	Chinese criteria	9.8 (2.9)	NA	NA	[35]
36	Mahlknecht, P.	2012	Austria	Serum	ELISA	20	30	67 (52-81)	65 (56-80)	M8 F12	M12 F18	NA	6 (1-37)	2 (1–4)	NA	[36]
37	Sathe, K.	2012	Britain	CSF/ Serum	ELISA	82	64	69 (8)	67 (9)	M40 F42	M23 F41	UKPDSBBC	8 (6)	2 (1–4)	NA	[37]
38	Ton, T. G.	2012	USA	Blood	ELISA	60	5674	NA	NA	NA	NA	Medical records	NA	NA	NA	[38]

39	Zhao, X. Q.	2012	China	Serum	ELISA	40	30	67.3 (9.4)	62.1 (10.4)	M23 F17	M17 F13	Chinese criteria	4.7 (3.8)	1-5	6.5 (9.7)	[39]
40	Lindqvist, D.	2013	Sweden	CSF	MSD	71	33	64.1 (10.5)	65.8 (8.8)	M14 F19	M44 F27	NA	6.4 (5.6)	1.9 (0.8)	18 (10-25)	[40]
41	Olsson, B.	2013	Sweden	CSF	ELISA	50	37	60.4 (9.6)	69.2 (7.3)	M36 F14	M13 F24	Gelb DJ's criteria	11.1 (7.4)	NA	NA	[41]
42	Rocha, N. P.	2014	Brazil	Plasma	ELISA	40	25	68.7 (10.1)	65.2 (8.8)	M27F 13	M19 F6	UKPDSBBC	5.5 (4.1)	2.4 (0.7)	34.6 (18.4)	[42]
43	Rocha, N. P.	2014	Brazil	Plasma	ELISA	40	25	68.7 (10.1)	65.2 (8.8)	M27 F13	M19 F6	UKPDSBBC	5.5 (4.1)	2.4 (0.7)	34.6 (18.4)	[43]
44	Song, I. U.	2014	Korea	Serum	NA	435	221	69.4 (9.2)	68.5 (11.5)	M147 F278	M95 F126	UKPDSBBC	3.3 (2.8)	2.2 (1.0)	NA	[44]
45	Sawada, H.	2014	Japan	Plasma	NA	111	-	69.7 (7.8)	-	M46 F65	-	UKPDSBBC	8.0 (4.5)	NA	22.2 (11.4)	[45]
46	Tang, P.	2014	China	Serum	ELISA	78	80	76.3 (5.0)	75.4 (4.4)	M60 F18	M59 F21	UKPDSBBC	5.5 (5.2)	2.6 (1.3)	27.2 (12.4)	[46]
47	Wang, Y. R.	2014	China	Serum	ELISA	56	120	69.4 (7.4)	69.5 (9.3)	M21 F35	M59 F61	UKPDSBBC	3 (3)	2 (1.5)	NA	[47]
48	Yu, S. Y.	2014	China	CSF	ELISA	26	31	57.4 (10.8)	52.2 (8.6)	M13 F13	M17 F14	UKPDSBBC	2 (2-5)	2 (1-3)	NA	[48]
49	Akıl, E.	2015	Turkey	Serum	Nephelometric	51	50	69.5 (9.3)	65.9 (10.1)	M29 F22	M26 F24	UKPDSBBC	NA	NA	NA	[49]
50	Bu, X. L.	2015	China	Serum	ELISA	131	141	67 (10)	68 (9)	M68 F63	M75 F66	UKPDSBBC	3.6 (3.4)	2.2 (0.9)	NA	[50]
51	Hu, Y.	2015	China	CSF	ELISA	84	31	NA	NA	NA	NA	UKPDSBBC	NA	NA	NA	[51]
52	Jiang, Q. W.	2015	China	Plasma	ELISA	59	54	64.4 (8.1)	65.0 (6.9)	M41	M37	UKPDSBBC	3.3 (3.1)	1.4 (0.5)	14.6 (7.2)	[52]

										F18	F17					
53	Magdalinou, N. K.	2015	Britain	CSF	MSD	31	30	67 (64-70)	60 (56-63)	M20 F11	M30 F30	NA	NA	NA	NA	[53]
54	Martín de Pablos, A.	2015	Spain	CSF	ELISA	37	21	63.4 (0.9)	67.9 (10)	M14 F15	M13 F8	NA	NA	1-4	NA	[54]
55	Park, S. J.	2015	Korea	Serum	NA	112	94	72.9 (5.7)	71.6 (7.9)	M36 F76	M45 F49	UKPDSBBC	2.6 (2.5)	NA	NA	[55]
56	Umemura, A.	2015	Japan	Serum	NA	375	65	69.3	NA	NA	NA	UKPDSBBC	NA	NA	NA	[56]
57	Wang, Y. H.	2015	China	Plasma	Nephelometric	62	38	65.8 (9.3)	69.4 (10.1)	M27 F35	M18 F20	UKPDSBBC	6.4 (1.2)	2.5 (0.8)	20.6 (6.8)	[57]
58	Wennstrom, M.	2015	USA	CSF	ELISA	61	-	68.4 (9.2)	-	M36F25	-	NINDS	NA	NA	NA	[58]
59	Csencsits-Smith, K.	2016	USA	Serum	Millipore	24	15	64.2 (8.6)	56.8 (9.2)	M16 F8	M10 F5	UKPDSBBC	5.2 (3.3)	1.6 (0.8)	29.1 (14.8)	[59]
60	da Silva, D. J.	2016	Brazil	Serum	CBA	21	21	59 (51-68)	59 (46-69)	M13 F8	M13 F8	UKPDSBBC	6 (1-20)	2 (2-3)	19 (9.5-40)	[60]
61	de Farias, C. C.	2016	Brazil	Blood	Nephelometric	56	54	70.3 (8.9)	69.7 (8.8)	M27 F29	M24 F30	UKPDSBBC	6.5 (4.2)	NA	NA	[61]
62	Gupta, V.	2016	India	Serum	ELISA	81	83	58.5 (8.8)	57.6 (7.9)	M60 F21	M59 F24	UKPDSBBC	3.2 (2.9)	NA	NA	[62]
63	Hall, S.	2016	Sweden	CSF	ELISA	63	21	64.7 (9.4)	65.7 (6.8)	M42 F21	M8 F13	NA	5.5 (4.0)	2.0 (0.7)	19.2 (9.4)	[63]
64	Li, Z. J.	2016	China	Serum	ELISA	65	52	64.6 (8.2)	NA	M36 F29	NA	UKPDSBBC	2.7 (1.0)	NA	20.1 (5.6)	[64]
65	Lue, L. F.	2016	USA	Plasma	Multiplex	74	-	73.1 (1.3)	-	M48 F26	-	Clinical	NA	NA	25.5 (9.5)	[65]

66	Pereira, J. R.	2016	Brazil	Serum	ELISA	44	-	65.1 (10.9)	-	M33 F11	-	UKPDSBBC	6.4 (3.7)	NA	35.8 (10.3)	[66]
67	Wang, X. M.	2016	China	Blood	ELISA	62	62	65.0 (7.2)	64.6 (7.5)	M38 F24	M36 F26	Tolosa E's criteria	NA	NA	NA	[67]
68	Williams-Gray, C. H.	2016	British	Serum	V-PLEX	230	-	66.4 (9.5)	-	M142 F78	-	UKPDSBBC	0.6 (0.5)	NA	27.9 (11.5)	[68]
69	Delgado-Alvarado, M.	2017	Spain	CSF/ Plasma	Luminex Xmap	39	38	71.3 (6.2)	68.1 (4.9)	M28 F11	M20 F18	MDS criteria	7.9 (4.1)	2.3 (0.5)	22.2 (7.9)	[69]
70	Eidson, L. N.	2017	USA	CSF/ Serum	MSD	12	6	51.8 (8.0)	53.3 (11.4)	M8 F4	M4 F2	NA	NA	NA	NA	[70]
71	Bagheri, V.	2018	Iran	Serum	ELISA	30	40	68.7 (4.1)	69.2 (9.3)	M19 F11	M26 F14	MDS criteria	4.2 (3.0)	NA	22.1 (8.6)	[71]
72	Dos Santos, M. C. T.	2018	Germany	CSF	Millipore	80	80	64.3 (9.8)	62.7 (10.2)	M53 F26	M51 F29	UKPDSBBC	NA	NA	NA	[72]
73	Hall, S.	2018	Sweden	CSF	MSD	131	50	64.9 (10.6)	65.3 (8.6)	M80 F51	M22 F28	NINDSDC	5.5 (4.8)	2.0 (0.8)	17.1 (10.5)	[73]
74	Ilyechova, E. Y.	2018	Russia	Serum	ELISA	50	50	64 (57-72)	60 (55-74)	M21 F29	M24 F26	UKPDSBBC	NA	3 (1.5-2.5)	28 (26-29)	[74]
75	Karpenko, M. N.	2018	Sweden	Serum	ELISA	117	60	65 (57-73)	65 (59-72)	M49 F68	M29 F31	Medical records	5 (2-16)	3 (1.5-2.5)	30 (25-41)	[75]
76	Kim, R.	2018	Korea	Serum	MSD	58	20	62.4 (8.1)	61.1 (10.3)	M27 F31	M9 F11	UKPDSBBC	1.2 (0.9)	1.8 (0.7)	26.7 (10.5)	[76]
77	Kouchaki, E.	2018	Iran	Serum	ELISA	43	40	65.1 (9.7)	66.7 (12.7)	M28 F15	M24 F16	UKPDSBBC	5.6 (4.4)	2.2 (0.7)	NA	[77]
78	Kouchaki, E.	2018	Iran	Serum	ELISA	83	83	65.7 (11.2)	64.2 (12.6)	M52 F31	M38 F45	UKPDSBBC	4.9 (3.9)	2.2 (0.7)	NA	[78]

79	Li, D. H.	2018	China	Blood	ELISA	23	24	NA	NA	NA	NA	MDS criteria	3.5 (3.3)	1.8 (0.7)	20.1 (13.3)	[79]
80	Moghaddam, H. S.	2018	Iran	CSF	NA	109	84	69.7 (6.5)	67.1 (7.2)	M66 F43	M39 F45	NA	NA	NA	NA	[80]
81	Pochmann, D.	2018	Brazil	Plasma	ELISA	12	14	65.1 (6.7)	60.2 (10.3)	M6 F6	M3 F11	NA	NA	NA	15.8 (7.5)	[81]
82	Rocha, N. P.	2018	Brazil	Plasma	CBA	40	25	68.7 (10.1)	65.2 (8.8)	M27 F13	M19 F6	UKPDSBBC	NA	2.4 (0.7)	34.6 (18.4)	[82]
83	Rocha Sobrinho, H. M. D.	2018	Brazil	Serum	NA	26	26	66 (35-76)	65 (34- 75)	M20 F6	M20 F6	UKPDSBBC	13 (5-20)	2 (2-3)	20 (19-46)	[83]
84	Solmaz, V.	2018	Turkey	Blood	Nephelometric	101	60	66.4 (10.9)	63.3 (8.9)	M58 F43	NA	UKPDSBBC	NA	NA	NA	[84]
85	Starhof, C.	2018	Denmark	CSF	MSD	46	31	64.5 (11.5)	45.5 (17.7)	M30 F16	M15 F16	NA	7.0 (3.8)	2 (2)	NA	[85]
86	Veselý, B.	2018	Slovak Republic	Serum	ECLIA	47	-	65 (7.8)	-	M28F19	-	UKPDSBBC	7.7 (4.2)	NA	NA	[86]
87	Yang, Y.	2018	China	Serum	ELISA	72	20	64.2 (5.4)	62.2 (3.8)	M38 F34	M10 F10	Chinese criteria	NA	NA	NA	[87]
88	Ahmadi Rastegar, D.	2019	Australia	Serum	Multiplex	65	-	NA	-	NA	-	UKPDSBBC	NA	NA	NA	[88]
89	Alrafiah, A.	2019	Arabia	Serum	Milliplex	26	24	60.4 (11.5)	60.2 (9.2)	M20 F6	M18 F6	NA	NA	NA	NA	[89]
90	Álvarez-Luquín, D. D.	2019	Mexico	Plasma	ELISA	32	22	60.8 (10.2)	55.6 (10.2)	NA	NA	UKPDSBBC	NA	NA	NA	[90]
91	Baran, A.	2019	Turkey	Serum	Nephelometric	30	30	70.7 (9.6)	66.8 (9.0)	M16 F14	M14 F16	UKPDSBBC	5.9 (4.7)	3.3 (1.3)	NA	[91]

92	Compta, Y.	2019	Spain	CSF	Luminex Xmap	17	14	65 (63-67)	68 (61-73)	M12 F7	M8 F7	NA	27 (18-33)	NA	NA	[92]
93	Green, H. F.	2019	Sweden	Plasma	SIMOA	63	43	69.9 (8.1)	68.2 (7.1)	M34 F32	M24 F21	NA	5 (0-26)	2 (1-5)	28.9 (13.3)	[93]
94	King, E.	2019	Britain	Serum	MSD	112	64	69.5 (6.7)	69.5 (0.8)	M44 F68	M34 F30	UKPDSBBC	NA	NA	22.8 (12.1)	[94]
95	Lian, T. H.	2019	China	CSF/ Serum	ELISA	113	35	59.2 (9.6)		M62 F51	NA	MDS criteria	NA	NA	NA	[95]
96	Lin, C. H.	2019	Taiwan	Plasma	Procarta Plex	120 /80	120 / 77	62.3 (7.8)/ 64.0 (8.8)	61.8 (8.3)/ 62.1 (4.7)	M75 F45/ M43 F37	M72 F48/ M36 F41	UKPDSBBC	7.5 (5.2)	1.8 (0.9)	17.4 (9.6)	[96]
97	Mollenhauer, B.	2019	Germany	Blood	NA	135	107	64.5 (9.8)	64.7 (6.9)	NA	NA	UKPDSBBC	NA	NA	22.8 (12.1)	[97]
98	Perner, C.	2019	Germany	Plasma	ELISA	33	33	69.6 (10.4)	63.7 (11.7)	M16 F17	M20 F13	UKPDSBBC	10.1 (5.1)	3 (1)	34.5 (15.1)	[98]
99	Santos-Garcia, D.	2019	Spain	Blood	ELISA	153	65	NA	60.3 (6.1)	NA	M37 F28	NA	NA	NA	NA	[99]
100	Yazar, T.	2019	Turkey	Serum	Nephelometric	151	150	72.6 (7.5)	73.9 (4.8)	NA	NA	UKPDSBBC	NA	NA	NA	[100]
101	Calvani, R.	2020	Italy	Serum	Luminex Xmap	20	30	73.1 (10.2)	74.6 (4.3)	M11 F9	M14 F16	UKPDSBBC	8.2 (5.8)	NA	NA	[101]
102	Chatterjee, K.	2020	India	Serum	ELISA	27	15	62.5 (7.7)	62.7 (6.9)	M14 F13	M10 F5	UKPDSBBC	6.7 (3.4)	2 (0.8)	25.8 (15.1)	[102]
103	Fan, Z	2020	China	Plasma	MSD	43	24	58.4 (1.4)	57.9 (1.6)	M24 F19	M13 F11	UKPDSBBC	2.3 (0.3)	1.9 (0.1)	31.4 (2.1)	[103]

104	Iwaoka, K.	2020	Japan	CSF	ELISA	20	13	69.0 (6.4)	69.0 (16.7)	M9 F11	M9 F4	UKPDSBBC	29.6 (20.3)	2.5 (0.5)	25.2 (9.5)	[104]
105	Jin, H.	2020	China	Serum	NA	183	88	59.7 (10.6)	62.1 (7.1)	NA	NA	UKPDSBBC	NA	2.0 (0.7)	24.3 (12.7)	[105]
106	Kiçik, A.	2020	Turkey	Serum	ELISA	61	-	62.6 (8.9)	-	M17 F37	-	UKPDSBBC	6.2 (3.6)	1.8 (0.5)	28.7 (11.6)	[106]
107	Kwiatek-Majkusiak, J.	2020	Poland	Serum	ELISA	47	28	60.2 (10.4)	58.4 (2.4)	M26 F21	M14 F14	UKPDSBBC	8.4 (7.7)	NA	12.6 (11.0)	[107]
108	Majbour, N. K.	2020	Norway	CSF	Luminex Xmap	60	43	57 (10)	49 (18)	M36 F24	M19 F24	NA	NA	NA	NA	[108]
109	Martin-Ruiz, C.	2020	Britain	Serum	MSD	154	99	67 (60-82)	68 (63-82)	M54 F100	M54 F45	UKPDSBBC	0.4 (0.2-1)	2 (2-3)	26 (17-47)	[109]
110	Miliukhina, I. V.	2020	Russia	Plasma	ELISA/Luminex Xmap	23/ 28	29/ 28	66.5 (2.3)/ 70.7 (1.6)	68.5 (2.9)/ 67.5 (1.4)	M10F13/ M12F16	M16 F13/ M15 F13	MDS criteria	NA	NA	NA	[110]
111	Moghadam-Ahmadi, A.	2020	Iran	Serum	ELISA	30	30	68.7 (4.1)	69.2 (9.3)	M19 F11	M26 F14	UKPDSBBC	4.3 (3.0)	NA	NA	[111]
112	Lian, T. H.	2020	China	CSF	ELISA	86	-	62.2 (9.5)	-	M46 F40	-	UKPDSBBC	3.2 (2.5)	NA	27.1 (16.2)	[112]
113	Peng, G.	2020	China	CSF/ Plasma	ELISA	55	40	59.8 (8.9)	55.6 (13.4)	M27 F28	M21 F19	UKPDSBBC	3.6 (1-15)	2.0 (1-3)	22.6 (12.2)	[113]
114	Sankowski, B.	2020	Poland	Serum	Nephelometric	18	9	59 (10)	42 (10)	M8 F10	M4 F5	UKPDSBBC	8.1 (4.4)	2.9 (0.6)	40 (18)	[114]
115	Santaella, A.	2020	Netherlands	CSF	Multiplex PEA	44	25	57.9 (9.9)	64.5 (10.3)	M28 F16	M11 F14	MDS criteria	3.5 (2.9)	2.0 (0.6)	28.2 (13.8)	[115]

116	Santaella, A.	2020	Netherlands	CSF	ELISA	46	-	57.5 (10.0)	-	M30 F16	-	MDS criteria	3.0 (0.8)	2.0 (0.7)	27.3 (12.7)	[116]
117	Szymura, J.	2020	Poland	Plasma	ELISA	29	32	65.7 (7.5)	66.5 (3.2)	M19 F19	M20 F12	NA	NA	2-3	NA	[117]
118	Usenko, T. S.	2020	Russia	Plasma	Milliplex	29	19	67 (45-73)	64 (35-86)	M14 F15	M6 F13	MDS criteria	NA	NA	NA	[118]
119	Yang, W. L.	2020	China	Plasma	Nephelometric	204	204	63.9 (11.2)	63.8 (11.4)	M92 F112	M92 F112	MDS criteria	4.8 (3.7)	2.4 (1.0)	21.3 (11.1)	[119]
120	Yu, C. C.	2020	Taiwan	Serum	ELISA	33	27	63.1 (9.1)	63.1 (9.1)	M11 F22	M7 F20	UKPDSBBC	24.7 (16.6)	1.7 (0.9)	NA	[120]
121	Bartl, M.	2021	USA	CSF	ELISA	252	115	61 (9.8)	62 (11)	M165 F87	M74 F41	MDS criteria	NA	NA	20 (8.5)	[121]
122	Chen, S. J.	2021	China	Plasma	ELISA	248	149	68.9 (10.8)	67.7 (9.9)	M115 F133	M69 F80	UKPDSBBC	6.0 (4.0)	2.2 (1.0)	21.6 (12.3)	[122]
123	Galper, J.	2021	USA	Plasma	Bio-Plex	75	77	62.4 (1.2)	62.6 (1.2)	M37 F38	M40 F37	NA	NA	NA	17.3 (1.2)	[123]
124	Hatcher-Martin, J. M.	2021	USA	CSF	Milliplex	19	12	70.4 (10.1)	74 (10.0)	M12 F7	M4 F8	UKPDSBBC	9.6 (4.2)	NA	23.8 (13.4)	[124]
125	Hu, Y.	2021	China	CSF/ Serum	ELISA	139	30	NA	NA	NA	NA	MDS criteria	NA	NA	NA	[125]
126	Li, S. Y.	2021	China	Serum	Nephelometry	148	-	63.8 (11.1)	-	M92F56	-	UKPDSBBC	4.9 (4.3)	NA	NA	[126]
127	Lin, Y. W.	2021	China	CSF/ Plasma	Nephelometric	250	250	63.2 (11.2)	63.3 (12.3)	M113 F87	M108 F92	MDS criteria	NA	2.3 (0.8)	33.1 (11.6)	[127]
128	Mo, M. S.	2021	China	CSF	ELISA	80	65	63.6 (8.5)	62.5 (6.9)	M48	M39	UKPDSBBC	3.2 (1.5)	2.0 (0.4)	30.4 (15.3)	[128]

										F32	F26					
129	Muruzheva, Z. M.	2021	Russia	Plasma	ELISA	12	12	63 (60-66)	63 (58-68)	M6 F6	M6 F6	UKPDSBBC	NA	2 (2-3)	NA	[129]
130	Roy, A.	2021	India	Serum	ELISA	20	15	63.13 (2.2)	62.7 (2.1)	M13 F7	M10 F5	NA	NA	NA	NA	[130]
131	Solini, A.	2021	Italy	Plasma	ELISA	25	25	69.2 (6.9)	69.1 (7.7)	M14 F11	M10 F15	UKPDSBBC	9.1 (3.2)	1.5 (0.6)	21.2 (9.0)	[131]
132	Thaler, A.	2021	Israel	Blood	NA	105	220	66.5 (10.5)	54.1 (11.3)	M72 F33	M102 F118	UKPDSBBC	2.6 (2.0)	NA	23.0 (12.0)	[132]
133	Wu, H. C.	2021	Taiwan	Plasma	ELISA	56	46	64.8 (10.2)	65.9 (9.8)	M32 F24	M25 F21	UKPDSBBC	6.0 (5.5)	2.6 (1.1)	NA	[133]
134	Wu, Z. B.	2021	China	Serum	NA	58	60	65.2 (10.2)	65.8 (4.9)	M29 F29	M33 F27	UKPDSBBC	3 (1.5-7.0)	2 (1.5-2.5)	NA	[134]
135	Xia, D. H.	2021	China	CSF	ELISA	34	29	61.8 (4.1)	60.5 (4.6)	M19 F15	M12 F17	MDS criteria	NA	2 (1.5-2.5）	NA	[135]
136	Yin, Y. H.	2021	China	Serum	ELISA	73	20	65.3 (5.3)	58.3 (6.3)	M45 F28	M12 F8	Chinese criteria	1-8	NA	NA	[136]
137	Zhu, Y.	2021	China	Serum	ELISA	46	42	69.5 (9.6)	69.7 (9.0)	M28 F18	M24 F18	EFNS and MDSES	3 (1-8)	2 (2-3)	26.4 (12.6)	[137]
138	Al-Huchaimi, S. H. K.	2022	Iraq	Serum	ELISA	35	45	55.5 (17.3)	52.3 (16.5)	M19 F16	M33 F12	NA	NA	NA	NA	[138]
139	Diaz, K.	2022	USA	Serum	Milliplex	26	14	72.8 (7.1)	69.2 (5.4)	M6 F8	M11 F15	NA	8.0 (5.4)	2.2 (0.5)	68.2 (17.1)	[139]
140	El-Kattan, M. M.	2022	Egypt	Serum	ELISA	30	30	63 (8)	62 (4)	M25 F5	M25 F5	MDS criteria	3 (1)	NA	NA	[140]

141	Ghit, A.	2022	Egypt	Serum	ELISA	20	15	61.7 (14.6)	61.8 (4.9)	M11 F9	M10 F5	UKPDSBBC	4.3 (4.0)	NA	NA	[141]
142	Gupta, M.	2022	India	Serum	ELISA	21	-	57.9 (9.3)	-	M14 F7	-	NA	3.0 (1.6)	NA	NA	[142]
143	Haji, S.	2022	Japan	Serum	U-PLEX	29	41	67 (60-71)	67 (53-70)	M17 F12	M17 F24	UKPDSBBC	43 (24-72)	2 (2-3)	NA	[143]
144	Imarisio, A.	2022	Italy	Plasma	Elecsys	71	69	65.1 (10.5)	71.7 (7.1)	M46 F25	M41 F28	NA	5.2 (7.0)	NA	16.6 (10.1)	[144]
145	Li, Y. Y.	2022	China	Plasma	MSD	76	76	62.2 (7.5)	60.2 (8.2)	M38 F38	M48 F28	MDS criteria	4.9 (4.3)	NA	28.4 (15.1)	[145]
146	Kaminska, M.	2022	Canada	Serum	Multiplex	66	-	64.6 (9.8)	-	M41 F25	-	NA	NA	NA	22.0 (12.7)	[146]
147	Liu, J.	2022	China	Plasma	Nephelom etric	145	72	64.9 (11.0)	68.8 (9.5)	M93 F52	M35 F37	MDS criteria	4.0 (3.5)	2.0 (0.92)	24.5 (13.7)	[147]
148	Lerche, S.	2022	Germany	CSF	Multiplex	68	-	NA	-	NA	-	UKPDSBBC	NA	NA	NA	[148]
149	Wang, L. X.	2022	China	Blood	NA	93	36	61 (51-68)	61 (57-67)	M60 F33	M19 F17	MDS criteria	NA	2(1-4)	20.2 (1.4)	[149]
150	Xu, J. H.	2022	China	Serum	Bio-Rad	32	30	61.2 (8.5)	55.8 (12.9)	M15 F17	M13 F17	MDS criteria	5.4 (4.0)	2.6 (0.9)	44.7 (20.4)	[150]
151	Yuan, Y.	2022	China	CSF/ Serum	Chemical	13	-	NA	-	NA	-	MDS criteria	NA	NA	NA	[151]
152	Zhao, Y.	2022	China	Plasma	ELISA	36	36	66.5 (1.37)	66.9 (1.5)	M16 F20	M18 F19	NA	5.8 (0.7)	NA	NA	[152]

References

1. Mogi, M., et al., *Tumor necrosis factor-alpha (TNF-alpha) increases both in the brain and in the cerebrospinal fluid from parkinsonian patients.* Neurosci Lett, 1994. **165**(1-2): p. 208-10.
2. BlumDegen, D., et al., *Interleukin-1 beta and interleukin-6 are elevated in the cerebrospinal fluid of Alzheimer's and de novo Parkinson's disease patients.* Neuroscience Letters, 1995. **202**(1-2): p. 17-20.
3. Klueter, H., et al., *Defective production of interleukin-2 in patients with idiopathic Parkinson's disease.* Journal of the Neurological Sciences, 1995. **133**(1-2): p. 134-139.
4. Mogi, M., et al., *Interleukin (IL)-1 beta, IL-2, IL-4, IL-6 and transforming growth factor-alpha levels are elevated in ventricular cerebrospinal fluid in juvenile parkinsonism and Parkinson's disease.* Neuroscience Letters, 1996. **211**(1): p. 13-16.
5. Stypula, G., et al., *Evaluation of interleukins, ACTH, cortisol and prolactin concentrations in the blood of patients with Parkinson's disease.* Neuroimmunomodulation, 1996. **3**(2-3): p. 131-134.
6. Vawter, M.P., et al., *TGF beta 1 and TGF beta 2 concentrations are elevated in Parkinson's disease in ventricular cerebrospinal fluid.* Experimental Neurology, 1996. **142**(2): p. 313-322.
7. Mueller, T., et al., *Interleukin-6 levels in cerebrospinal fluid inversely correlate to severity of Parkinson's disease.* Acta Neurologica Scandinavica, 1998. **98**(2): p. 142-144.
8. Dobbs, R.J., et al., *Association of circulating TNF-alpha and IL-6 with ageing and parkinsonism.* Acta Neurologica Scandinavica, 1999. **100**(1): p. 34-41.
9. Wandinger, K.P., et al., *Effects of amantadine treatment on in vitro production of interleukin-2 in de-novo patients with idiopathic Parkinson's disease.* Journal Of Neuroimmunology, 1999. **98**(2): p. 214-220.
10. Yang, Z.Z., P.L. Geng, and R.Y. Wang, *The concentration of IL-1beta, TNF-alpha and nitric oxide in serum and cerebrospinal fluid in the patients with some kind of CNS diseases and their significance.* Xi'an Yike Daxue Xuebao, 2001. **22**(1): p. 43-47.
11. Selikhova, M.V., et al., *Impaired production of plasma interleukin-6 in patients with Parkinson's disease.* Bulletin Of Experimental Biology And Medicine, 2002. **133**(1): p. 81-83.
12. Rota, E., et al., *Increased intrathecal TGF-β1, but not IL-12, IFN-γ and IL-10 levels in Alzheimer's disease patients.* Neurological Sciences, 2006. **27**(1): p. 33-39.
13. Rentzos, M., et al., *Circulating interleukin-15 and RANTES chemokine in Parkinson's disease.* Acta Neurologica Scandinavica, 2007. **116**(6): p. 374-379.
14. Brodacki, B.D., et al., *Serum interleukin (IL-2, IL-10, IL-6, IL-4), TNF alpha, and INF gamma concentrations are elevated in patients with atypical and idiopathic parkinsonism.* Neuroscience Letters, 2008. **441**(2): p. 158-162.

15. Chen, H.L., et al., *Peripheral inflammatory biomarkers and risk of Parkinson's disease.* American Journal Of Epidemiology, 2008. **167**(1): p. 90-95.
16. Choi, C., et al., *Multiplex analysis of cytokines in the serum and cerebrospinal fluid of patients with Alzheimer's disease by color-coded bead technology.* J Clin Neurol, 2008. **4**(2): p. 84-8.
17. P., X.J., et al., *The research on inflammatory factors of serum in the patient with Parkinson's disease.* Chinese Journal of Immunology, 2008. **24**(3): p. 266-268,270.
18. Dufek, M., et al., *Serum inflammatory biomarkers in Parkinson's disease.* Parkinsonism & Related Disorders, 2009. **15**(4): p. 318-320.
19. Hofmann, K.W., et al., *Interleukin-6 serum levels in patients with Parkinson's disease.* Neurochem Res, 2009. **34**(8): p. 1401-4.
20. Rentzos, M., et al., *Circulating interleukin-10 and interleukin-12 in Parkinson's disease.* Acta Neurologica Scandinavica, 2009. **119**(5): p. 332-337.
21. Scalzo, P., et al., *Increased serum levels of soluble tumor necrosis factor-alpha receptor-1 in patients with Parkinson's disease.* Journal Of Neuroimmunology, 2009. **216**(1-2): p. 122-125.
22. Tuncel, D., F. Inanc Tolun, and I. Toru, *Serum insulin-like growth factor-1 and nitric oxide levels in parkinson's disease.* Mediators of Inflammation, 2009. **2009**.
23. Menza, M., et al., *The Role of Inflammatory Cytokines in Cognition and Other Non-Motor Symptoms of Parkinson's Disease.* Psychosomatics, 2010. **51**(6): p. 474-479.
24. Pålhagen, S., et al., *Monoamines, BDNF, IL-6 and corticosterone in CSF in patients with Parkinson's disease and major depression.* J Neurol, 2010. **257**(4): p. 524-32.
25. Scalzo, P., et al., *Serum levels of interleukin-6 are elevated in patients with Parkinson's disease and correlate with physical performance.* Neurosci Lett, 2010. **468**(1): p. 56-8.
26. Varani, K., et al., *A2A adenosine receptor overexpression and functionality, as well as TNF-alpha levels, correlate with motor symptoms in Parkinson's disease.* Faseb j, 2010. **24**(2): p. 587-98.
27. Hassin-Baer, S., et al., *Is C-reactive protein level a marker of advanced motor and neuropsychiatric complications in Parkinson's disease?* J Neural Transm (Vienna), 2011. **118**(4): p. 539-43.
28. Lee, H.W., J. Choi, and K. Suk, *Increases of pentraxin 3 plasma levels in patients with Parkinson's disease.* Mov Disord, 2011. **26**(13): p. 2364-70.
29. Scalzo, P., et al., *Serum levels of chemokines in Parkinson's disease.* Neuroimmunomodulation, 2011. **18**(4): p. 240-4.
30. Shi, M., et al., *Cerebrospinal Fluid Biomarkers for Parkinson Disease Diagnosis and Progression.* Annals Of Neurology, 2011. **69**(3): p. 570-580.
31. Andican, G., et al., *Plasma oxidative and inflammatory markers in patients with idiopathic Parkinson's disease.* Acta Neurologica Belgica, 2012. **112**(2): p. 155-159.
32. Gruden, M.A., et al., *Correlation between protective immunity to α-synuclein aggregates, oxidative stress and inflammation.* Neuroimmunomodulation, 2012. **19**(6): p. 334-42.

33. Koziorowski, D., et al., *Inflammatory cytokines and NT-proCNP in Parkinson's disease patients.* Cytokine, 2012. **60**(3): p. 762-766.
34. Lindqvist, D., et al., *Non-Motor Symptoms in Patients with Parkinson's Disease - Correlations with Inflammatory Cytokines in Serum.* Plos One, 2012. **7**(10).
35. Liu, J., N.W. Yu, and X.J. Li, *Changes of humoral immune function in patients with Parkinson disease.* Journal of Clinical Neurology (China), 2012. **25**(4): p. 293-295.
36. Mahlknecht, P., et al., *An antibody microarray analysis of serum cytokines in neurodegenerative Parkinsonian syndromes.* Proteome Science, 2012. **10**.
37. Sathe, K., et al., *S100B is increased in Parkinson's disease and ablation protects against MPTP-induced toxicity through the RAGE and TNF-α pathway.* Brain, 2012. **135**(Pt 11): p. 3336-47.
38. Ton, T.G., et al., *Markers of inflammation in prevalent and incident Parkinson's disease in the Cardiovascular Health Study.* Parkinsonism Relat Disord, 2012. **18**(3): p. 274-8.
39. Zhao, X.Q., P. Niu, and C. Su, *The Parkinson patients serum levels of TNF-alpha,sTNFR1,sTNFR2 detection and clinical significance.* Journal of Apoplexy and Nervous Diseases, 2012. **29**(10): p. 900-903.
40. Lindqvist, D., et al., *Cerebrospinal fluid inflammatory markers in Parkinson's disease - Associations with depression, fatigue, and cognitive impairment.* Brain Behavior And Immunity, 2013. **33**: p. 183-189.
41. Olsson, B., et al., *The glial marker YKL-40 is decreased in synucleinopathies.* Mov Disord, 2013. **28**(13): p. 1882-5.
42. Rocha, N.P., et al., *Cognitive Status Correlates with CXCL10/IP-10 Levels in Parkinson's Disease.* Parkinsons Disease, 2014. **2014**.
43. Rocha, N.P., et al., *Plasma levels of soluble tumor necrosis factor receptors are associated with cognitive performance in Parkinson's disease.* Movement Disorders, 2014. **29**(4): p. 527-531.
44. Song, I.U., et al., *Serum hs-CRP levels are increased in de Novo Parkinson's disease independently from age of onset.* Eur Neurol, 2014. **72**(5-6): p. 285-9.
45. Sawada, H., et al., *Subclinical Elevation of Plasma C-Reactive Protein and Illusions/Hallucinations in Subjects with Parkinson's Disease: Case-control Study.* Plos One, 2014. **9**(1).
46. Tang, P., et al., *Correlation between Serum RANTES Levels and the Severity of Parkinson's Disease.* Oxidative Medicine And Cellular Longevity, 2014. **2014**: p. 1-4.
47. Wang, Y.R., et al., *Serum levels of inflammatory cytokines in Parkinson's disease and their clinical significance.* Chongqing Medicine, 2014. **43**(15): p. 1848-1850.
48. Yu, S.Y., et al., *Potential biomarkers relating pathological proteins, neuroinflammatory factors and free radicals in PD patients with cognitive impairment: a cross-sectional study.* BMC Neurol, 2014. **14**: p. 113.
49. Akıl, E., et al., *The increase of carcinoembryonic antigen (CEA), high-sensitivity C-reactive protein, and neutrophil/lymphocyte ratio in Parkinson's disease.* Neurol Sci, 2015. **36**(3): p. 423-8.

50. Bu, X.L., et al., *The association between infectious burden and Parkinson's disease: A case-control study.* Parkinsonism Relat Disord, 2015. **21**(8): p. 877-81.
51. Hu, Y., et al., *Parkinson disease with REM sleep behavior disorder: features, α-synuclein, and inflammation.* Neurology, 2015. **84**(9): p. 888-94.
52. Jiang, Q.W., J.F. Ma, and S.D. Chen, *Correlation between plasma macrophage inflammatory protein and early Parkinson's disease.* Chinese Journal of Neurology, 2015. **48**(6): p. 464-468.
53. Magdalinou, N.K., et al., *A panel of nine cerebrospinal fluid biomarkers may identify patients with atypical parkinsonian syndromes.* J Neurol Neurosurg Psychiatry, 2015. **86**(11): p. 1240-7.
54. Martín de Pablos, A., J.M. García-Moreno, and E. Fernández, *Does the Cerebrospinal Fluid Reflect Altered Redox State But Not Neurotrophic Support Loss in Parkinson's Disease?* Antioxid Redox Signal, 2015. **23**(11): p. 893-8.
55. Park, S.J. and Y.S. Yang, *Clinical Implication of High Sensitivity C-Reactive Protein for the Development of Dementia in Parkinson’s Disease.* Dementia and Neurocognitive Disorders, 2015. **14**(3): p. 123-127.
56. Umemura, A., et al., *Baseline Plasma C-Reactive Protein Concentrations and Motor Prognosis in Parkinson Disease.* Plos One, 2015. **10**(8).
57. Wang, Y.H. and X.H. Shi, *A study on the association between the inflammatory factors and hallucination in patients with Parkinson disease.* Chinese Journal of Nervous and Mental Diseases, 2015. **41**(1): p. 1-4.
58. Wennstrom, M., et al., *The Inflammatory Marker YKL-40 Is Elevated in Cerebrospinal Fluid from Patients with Alzheimer's but Not Parkinson's Disease or Dementia with Lewy Bodies.* Plos One, 2015. **10**(8).
59. Csencsits-Smith, K., et al., *Serum Lymphocyte-Associated Cytokine Concentrations Change More Rapidly over Time in Multiple System Atrophy Compared to Parkinson Disease.* Neuroimmunomodulation, 2016. **23**(5-6): p. 301-308.
60. da Silva, D.J., et al., *Decreased Toll-Like Receptor 2 and Toll-Like Receptor 7/8-Induced Cytokines in Parkinson's Disease Patients.* Neuroimmunomodulation, 2016. **23**(1): p. 58-66.
61. de Farias, C.C., et al., *Highly specific changes in antioxidant levels and lipid peroxidation in Parkinson's disease and its progression: Disease and staging biomarkers and new drug targets.* Neurosci Lett, 2016. **617**: p. 66-71.
62. Gupta, V., R.K. Garg, and S. Khattri, *Levels of IL-8 and TNF-α decrease in Parkinson's disease.* Neurol Res, 2016. **38**(2): p. 98-102.
63. Hall, S., et al., *Longitudinal Measurements of Cerebrospinal Fluid Biomarkers in Parkinson's Disease.* Mov Disord, 2016. **31**(6): p. 898-905.
64. Li, Z.J., W. Zou, and Y. Yang, *Correlation of Serum Inflammatory Factors with Depression in Patients with Parkinson's Disease.* Acta Medicinae Universitatis Scientiae et Technologiae Huazhong, 2016. **45**(6): p. 593-596.
65. Lue, L.F., et al., *Converging mediators from immune and trophic pathways to identify Parkinson disease dementia.* Neurol Neuroimmunol Neuroinflamm, 2016. **3**(1):

p. e193.
66. Pereira, J.R., et al., *IL-6 serum levels are elevated in Parkinson's disease patients with fatigue compared to patients without fatigue.* J Neurol Sci, 2016. **370**: p. 153-156.
67. Wang, X.M., et al., *Relationship between levels of inflammatory cytokines in the peripheral blood and the severity of depression and anxiety in patients with Parkinson's disease.* European Review For Medical And Pharmacological Sciences, 2016. **20**(18): p. 3853-3856.
68. Williams-Gray, C.H., et al., *Serum immune markers and disease progression in an incident Parkinson's disease cohort (ICICLE-PD).* Mov Disord, 2016. **31**(7): p. 995-1003.
69. Delgado-Alvarado, M., et al., *Tau/alpha-Synuclein Ratio and Inflammatory Proteins in Parkinson's Disease: An Exploratory Study.* Movement Disorders, 2017. **32**(7): p. 1066-1073.
70. Eidson, L.N., et al., *Candidate inflammatory biomarkers display unique relationships with alpha-synuclein and correlate with measures of disease severity in subjects with Parkinson's disease.* Journal Of Neuroinflammation, 2017. **14**.
71. Bagheri, V., et al., *CXCL12 and CXCR4 in the Peripheral Blood of Patients with Parkinson's Disease.* Neuroimmunomodulation, 2018. **25**(4): p. 201-205.
72. Dos Santos, M.C.T., et al., *Evaluation of cerebrospinal fluid proteins as potential biomarkers for early stage Parkinson's disease diagnosis.* PLoS ONE, 2018. **13**(11).
73. Hall, S., et al., *Cerebrospinal fluid concentrations of inflammatory markers in Parkinson's disease and atypical parkinsonian disorders.* Sci Rep, 2018. **8**(1): p. 13276.
74. Ilyechova, E.Y., et al., *A low blood copper concentration is a co-morbidity burden factor in Parkinson's disease development.* Neurosci Res, 2018. **135**: p. 54-62.
75. Karpenko, M.N., et al., *Interleukin-1 beta, interleukin-1 receptor antagonist, interleukin-6, interleukin-10, and tumor necrosis factor-alpha levels in CSF and serum in relation to the clinical diversity of Parkinson's disease.* Cellular Immunology, 2018. **327**: p. 77-82.
76. Kim, R., et al., *Peripheral blood inflammatory markers in early Parkinson's disease.* J Clin Neurosci, 2018. **58**: p. 30-33.
77. Kouchaki, E., et al., *Correlation of serum levels and gene expression of tumor necrosis factor-α-induced protein-8 like-2 with Parkinson disease severity.* Metab Brain Dis, 2018. **33**(6): p. 1955-1959.
78. Kouchaki, E., et al., *Increased serum levels of TNF-α and decreased serum levels of IL-27 in patients with Parkinson disease and their correlation with disease severity.* Clin Neurol Neurosurg, 2018. **166**: p. 76-79.
79. Li, D., et al., *Association of Parkinson's disease-related pain with plasma interleukin-1, interleukin-6, interleukin-10, and tumour necrosis factor-alpha.* Neuroscience Letters, 2018. **683**: p. 181-184.
80. Moghaddam, H.S., et al., *Cerebrospinal Fluid C-Reactive Protein in Parkinson's Disease: Associations with Motor and Non-motor Symptoms.* Neuromolecular Medicine, 2018. **20**(3): p. 376-385.

81. Pochmann, D., et al., *Cytokine modulation in response to acute and chronic aquatic therapy intervention in Parkinson disease individuals: A pilot study.* Neurosci Lett, 2018. **674**: p. 30-35.
82. Rocha, N.P., et al., *Reduced Activated T Lymphocytes (CD4+CD25+) and Plasma Levels of Cytokines in Parkinson's Disease.* Mol Neurobiol, 2018. **55**(2): p. 1488-1497.
83. Rocha Sobrinho, H.M.D., et al., *TLR4 and TLR2 activation is differentially associated with age during Parkinson's disease.* Immunol Invest, 2018. **47**(1): p. 71-88.
84. Solmaz, V., et al., *Serum neutrophil-lymphocyte ratios, C-reactive protein and sedimentation levels in Parkinson's disease.* Cukurova Medical Journal, 2018. **43**(2): p. 305-311.
85. Starhof, C., et al., *Cerebrospinal fluid pro-inflammatory cytokines differentiate parkinsonian syndromes.* Journal Of Neuroinflammation, 2018. **15**.
86. Veselý, B., et al., *Interleukin 6 and complement serum level study in Parkinson's disease.* J Neural Transm (Vienna), 2018. **125**(5): p. 875-881.
87. Yang, Y., et al., *Helper T cell Th9 and its cytokine interleukin-9 in peripheral blood of patients with Parkinson's disease.* Chinese Journal of Neurology, 2018. **51**(3): p. 171-174.
88. Ahmadi Rastegar, D., et al., *Parkinson's progression prediction using machine learning and serum cytokines.* NPJ Parkinsons Dis, 2019. **5**: p. 14.
89. Alrafiah, A., et al., *Assessment of the Levels of Level of Biomarkers of Bone Matrix Glycoproteins and Inflammatory Cytokines from Saudi Parkinson Patients.* BioMed Research International, 2019. **2019**.
90. Álvarez-Luquín, D.D., et al., *Regulatory impairment in untreated Parkinson's disease is not restricted to Tregs: other regulatory populations are also involved.* J Neuroinflammation, 2019. **16**(1): p. 212.
91. Baran, A., et al., *High-sensitivity C-reactive protein and high mobility group box-1 levels in Parkinson's disease.* Neurological Sciences, 2019. **40**(1): p. 167-173.
92. Compta, Y., et al., *Cerebrospinal fluid cytokines in multiple system atrophy: A cross-sectional Catalan MSA registry study.* Parkinsonism Relat Disord, 2019. **65**: p. 3-12.
93. Green, H.F., S. Khosousi, and P. Svenningsson, *Plasma IL-6 and IL-17A Correlate with Severity of Motor and Non-Motor Symptoms in Parkinson's Disease.* Journal Of Parkinsons Disease, 2019. **9**(4): p. 705-709.
94. King, E., et al., *Inflammation in mild cognitive impairment due to Parkinson's disease, Lewy body disease, and Alzheimer's disease.* Int J Geriatr Psychiatry, 2019. **34**(8): p. 1244-1250.
95. Lian, T.H., et al., *Tremor-Dominant in Parkinson Disease: The Relevance to Iron Metabolism and Inflammation.* Front Neurosci, 2019. **13**: p. 255.
96. Lin, C.H., et al., *Altered gut microbiota and inflammatory cytokine responses in patients with Parkinson's disease.* J Neuroinflammation, 2019. **16**(1): p. 129.
97. Mollenhauer, B., et al., *Baseline predictors for progression 4 years after Parkinson's disease diagnosis in the De Novo Parkinson Cohort (DeNoPa).* Mov Disord,

2019. **34**(1): p. 67-77.
98. Perner, C., et al., *Plasma VCAM1 levels correlate with disease severity in Parkinson's disease*. Journal Of Neuroinflammation, 2019. **16**.
99. Santos-Garcia, D., et al., *High ultrasensitive serum C-reactive protein may be related to freezing of gait in Parkinson's disease patients*. Journal Of Neural Transmission, 2019. **126**(12): p. 1599-1608.
100. Yazar, T. and H.O. Yazar, *Evaluation of C-reactive Protein/Albumin Ratio According to Stage in Patients with Idiopathic Parkinson Disease*. Turkish Journal Of Neurology, 2019. **25**(3): p. 123-128.
101. Calvani, R., et al., *A novel multi-marker discovery approach identifies new serum biomarkers for Parkinson's disease in older people: an EXosomes in PArkiNson Disease (EXPAND) ancillary study*. Geroscience, 2020. **42**(5): p. 1323-1334.
102. Chatterjee, K., et al., *Inflammasome and α-synuclein in Parkinson's disease: A cross-sectional study*. J Neuroimmunol, 2020. **338**: p. 577089.
103. Fan, Z., et al., *Systemic activation of NLRP3 inflammasome and plasma α-synuclein levels are correlated with motor severity and progression in Parkinson's disease*. J Neuroinflammation, 2020. **17**(1): p. 11.
104. Iwaoka, K., et al., *Impaired metabolism of kynurenine and its metabolites in CSF of parkinson's disease*. Neuroscience Letters, 2020. **714**.
105. Jin, H., et al., *Association of inflammatory factors and aging in Parkinson's disease*. Neurosci Lett, 2020. **736**: p. 135259.
106. Kiçik, A., et al., *Neuroinflammation mediators are reduced in sera of Parkinson's disease patients with mild cognitive impairment*. Noropsikiyatri Arsivi, 2020. **57**(1): p. 15-17.
107. Kwiatek-Majkusiak, J., et al., *Serum levels of hepcidin and interleukin 6 in Parkinson's disease*. Acta Neurobiologiae Experimentalis, 2020. **80**(3): p. 297-304.
108. Majbour, N.K., et al., *CSF total and oligomeric alpha-Synuclein along with TNF-alpha as risk biomarkers for Parkinson's disease: a study in LRRK2 mutation carriers*. Translational Neurodegeneration, 2020. **9**(1).
109. Martin-Ruiz, C., et al., *Senescence and Inflammatory Markers for Predicting Clinical Progression in Parkinson's Disease: The ICICLE-PD Study*. Journal Of Parkinsons Disease, 2020. **10**(1): p. 193-206.
110. Miliukhina, I.V., et al., *Plasma Cytokines Profile in Patients with Parkinson's Disease Associated with Mutations in GBA Gene*. Bulletin Of Experimental Biology And Medicine, 2020. **168**(4): p. 423-426.
111. Moghadam-Ahmadi, A., et al., *Eotaxins and C–C chemokine receptor type 3 in Parkinson's disease*. Acta Neurologica Belgica, 2020. **120**(3): p. 589-594.
112. Lian, T.H., et al., *Parkinson's Disease With Depression: The Correlations Between Neuroinflammatory Factors and Neurotransmitters in Cerebrospinal Fluid*. Front Aging Neurosci, 2020. **12**: p. 574776.
113. Peng, G., et al., *Analysis of Cerebrospinal Fluid Soluble TREM2 and Polymorphisms in Sporadic Parkinson's Disease in a Chinese Population*. J Mol Neurosci, 2020.

70(2): p. 294-301.
114. Sankowski, B., et al., *Higher cerebrospinal fluid to plasma ratio of p-cresol sulfate and indoxyl sulfate in patients with Parkinson's disease.* Clin Chim Acta, 2020. **501**: p. 165-173.
115. Santaella, A., et al., *Inflammation biomarker discovery in Parkinson's disease and atypical parkinsonisms.* Bmc Neurology, 2020. **20**(1).
116. Santaella, A., et al., *Cerebrospinal fluid monocyte chemoattractant protein 1 correlates with progression of Parkinson's disease.* NPJ Parkinson's disease, 2020. **6**: p. 21-21.
117. Szymura, J., et al., *The Immunomodulary Effects of Systematic Exercise in Older Adults and People with Parkinson's Disease.* Journal Of Clinical Medicine, 2020. **9**(1).
118. Usenko, T.S., et al., *Plasma cytokine profile in synucleinophaties with dementia.* Journal Of Clinical Neuroscience, 2020. **78**: p. 323-326.
119. Yang, W.L., et al., *Contra-Directional Expression of Plasma Superoxide Dismutase with Lipoprotein Cholesterol and High-Sensitivity C-reactive Protein as Important Markers of Parkinson's Disease Severity.* Frontiers In Aging Neuroscience, 2020. **12**.
120. Yu, C.C., et al., *Vascular Inflammation Is a Risk Factor Associated with Brain Atrophy and Disease Severity in Parkinson's Disease: A Case-Control Study.* Oxid Med Cell Longev, 2020. **2020**: p. 2591248.
121. Bartl, M., et al., *Biomarkers of neurodegeneration and glial activation validated in Alzheimer's disease assessed in longitudinal cerebrospinal fluid samples of Parkinson's disease.* PLoS One, 2021. **16**(10): p. e0257372.
122. Chen, S.J., et al., *Plasma Lipopolysaccharide-Binding Protein Reflects Risk and Progression of Parkinson's Disease.* J Parkinsons Dis, 2021. **11**(3): p. 1129-1139.
123. Galper, J., et al., *Cytokines and Gaucher Biomarkers in Glucocerebrosidase Carriers With and Without Parkinson Disease.* Movement Disorders, 2021. **36**(6): p. 1451-1455.
124. Hatcher-Martin, J.M., et al., *Cerebrospinal fluid biomarkers in Parkinson's disease with freezing of gait: an exploratory analysis.* Npj Parkinsons Disease, 2021. **7**(1).
125. Hu, Y., et al., *Clinical Characteristics, Iron Metabolism and Neuroinflammation: New Insight into Excessive Daytime Sleepiness in Parkinson's Disease.* Neuropsychiatr Dis Treat, 2021. **17**: p. 2041-2051.
126. Li, S.Y., et al., *Serum Folate, Vitamin B12 Levels, and Systemic Immune-Inflammation Index Correlate With Motor Performance in Parkinson's Disease: A Cross-Sectional Study.* Frontiers In Neurology, 2021. **12**.
127. Lin, Y.W., et al., *Bone-Derived Factors as Potential Biomarkers for Parkinson's Disease.* Frontiers In Aging Neuroscience, 2021. **13**.
128. Mo, M.S., et al., *Soluble Triggering Receptor Expressed on Myeloid Cells 2 From Cerebrospinal Fluid in Sleep Disorders Related to Parkinson's Disease.* Frontiers In Aging Neuroscience, 2021. **13**.

129. Muruzheva, Z.M., et al., *Calpain activity in plasma of patients with essential tremor and Parkinson's disease: a pilot study.* Neurological Research, 2021. **43**(4): p. 314-320.
130. Roy, A., et al., *Soluble LAG-3 and Toll-interacting protein: Novel upstream neuro-inflammatory markers in Parkinson's disease.* Parkinsonism & Related Disorders, 2021. **91**: p. 121-123.
131. Solini, A., et al., *P2X7 receptor/NLRP3 inflammasome complex and alpha-synuclein in peripheral blood mononuclear cells: a prospective study in neo-diagnosed, treatment-naive Parkinson's disease.* European Journal Of Neurology, 2021. **28**(8): p. 2648-2656.
132. Thaler, A., et al., *Biochemical markers for severity and risk in GBA and LRRK2 Parkinson's disease.* Journal Of Neurology, 2021. **268**(4): p. 1517-1525.
133. Wu, H.C., et al., *Alterations of plasma galectin-3 and c3 levels in patients with parkinson's disease.* Brain Sciences, 2021. **11**(11).
134. Wu, Z.B., et al., *Lipoprotein-Associated Phospholipase A2 Is a Risk Factor for Patients With Parkinson's Disease.* Frontiers In Neuroscience, 2021. **15**.
135. Xia, D.H., et al., *The evaluation of cerebrospinal fluid cytokines in the differential diagnosis of Parkinson disease and atypical parkinsonism.* Chinese Journal of Nervous and Mental Diseases, 2021. **47**(6): p. 355-360.
136. Yin, Y.H., X. Jin, and G.H. Wang, *Level and significance of follicular helper T cells (Tfh) and activated B cells in peripheral blood of patients with Parkinson ' s disease.* Chinese Journal of Immunology, 2021. **37**(6): p. 725-728.
137. Zhu, Y., et al., *Levels of serum IL6, TNF-α and sLAG3 are changed and correlated with clinical characteristics in Parkinson's disease patients: A case-control study.* Neurology Asia, 2021. **26**(4): p. 709-714.
138. Al-Huchaimi, S.H.K., G.H.N. Al-Kefaei, and B.A. Alameedy, *Effects of Toxoplasma gondii on Levels of Interleukin-5 in Parkinson's Patients.* Archives of Razi Institute, 2022. **77**(1): p. 195-202.
139. Diaz, K., et al., *Peripheral inflammatory cytokines and motor symptoms in persons with Parkinson's disease.* Brain, behavior, & immunity - health, 2022. **21**: p. 100442-100442.
140. El-Kattan, M.M., et al., *Relation of serum level of tumor necrosis factor-alpha to cognitive functions in patients with Parkinson's disease.* Egyptian Journal Of Neurology Psychiatry And Neurosurgery, 2022. **58**(1).
141. Ghit, A. and H.E. Deeb, *Cytokines, miRNAs, and Antioxidants as Combined Non-invasive Biomarkers for Parkinson's Disease.* J Mol Neurosci, 2022. **72**(5): p. 1133-1140.
142. Gupta, M., V.K. Paliwal, and G.N. Babu, *Serum fractalkine and 3-nitrotyrosine levels correlate with disease severity in Parkinson's disease: a pilot study.* Metabolic Brain Disease, 2022. **37**(1): p. 209-217.
143. Haji, S., et al., *Serum NfL and CHI3L1 for ALS and parkinsonian disorders in the process of diagnosis.* J Neural Transm (Vienna), 2022. **129**(3): p. 301-309.

144. Imarisio, A., et al., *Plasma Cystatin C correlates with plasma NfL levels and predicts disease progression in Parkinson's disease.* Neuro-degenerative diseases, 2022.
145. Li, Y.Y., et al., *Parkinson's disease peripheral immune biomarker profile: a multicentre, cross-sectional and longitudinal study.* Journal Of Neuroinflammation, 2022. **19**(1).
146. Kaminska, M., et al., *Inflammatory markers and BDNF in obstructive sleep apnea (OSA) in Parkinson's disease (PD).* Sleep medicine, 2022. **90**: p. 258-261.
147. Liu, J., et al., *Association between levels of high-sensitivity C-reactive protein in plasma and freezing of gait in Parkinson's disease.* Aging Clinical And Experimental Research, 2022.
148. Lerche, S., et al., *CSF and Serum Levels of Inflammatory Markers in PD: Sparse Correlation, Sex Differences and Association With Neurodegenerative Biomarkers.* Frontiers In Neurology, 2022. **13**.
149. Wang, L.X., et al., *Peripheral blood inflammatory cytokines are associated with rapid eye movement sleep behavior disorder in Parkinson's disease.* Neurosci Lett, 2022. **782**: p. 136692.
150. Xu, J.H., et al., *Characteristics of systemic inflammation and brain iron deposition in Parkinson's disease patients.* Annals Of Clinical And Translational Neurology, 2022. **9**(3): p. 276-285.
151. Yuan, Y., et al., *Cerebrospinal Fluid TNF-α and Orexin in Patients With Parkinson's Disease and Rapid Eye Movement Sleep Behavior Disorder.* Front Neurol, 2022. **13**: p. 826013.
152. Zhao, Y., et al., *Plasma proteome profiling using tandem mass tag labeling technology reveals potential biomarkers for Parkinson's disease: a preliminary study.* Proteomics Clin Appl, 2022. **16**(2): p. e2100010.

Supplementary Table 4. Quality assessment.

No.	Author	Year	*SELECTION*				*COMPARABILITY*	*EXPOSURE*			Total
			**1**	**2**	**3**	**4**	**1**	**1**	**2**	**3**	
1	Mogi, M.	1994		★		★	★	★	★	★	6
2	BlumDegen, D.	1995	★	★		★	★	★	★	★	7
3	Klueter, H.	1995	★	★	★	★	★	★	★	★	8
4	Mogi, M.	1996		★		★	★	★	★	★	6
5	Stypula, G.	1996		★		★	★★	★	★	★	7
6	Vawter, M. P.	1996		★		★	★	★	★	★	6
7	Mueller, T.	1998		★		★	★	★	★	★	6
8	Dobbs, R. J.	1999	★	★	★	★	★★	★	★	★	9
9	Wandinger, K. P.	1999	★	★		★	★★	★	★	★	8
10	Yang, Z. Z.	2001	★	★	★	★	★	★	★	★	8
11	Selikhova, M. V.	2002	★	★	★	★	★	★	★	★	8
12	Rota, E.	2006	★	★		★	★★	★	★	★	8
13	Rentzos, M.	2007	★	★	★	★	★★	★	★	★	9
14	Brodacki, B.	2008	★	★	★	★	★	★	★	★	8
15	Chen, H. L.	2008		★	★	★	★	★	★	★	7
16	Choi, C.	2008	★	★	★	★	★★	★	★	★	9
17	Xu. J. P.	2008	★	★	★	★	★	★	★	★	8
18	Dufek, M.	2009	★	★		★	★★	★	★		7
19	Hofmann, K. W.	2009	★	★	★	★	★★	★	★	★	9
20	Rentzos, M.	2009	★	★	★	★	★★	★	★	★	9
21	Scalzo, P.	2009		★		★	★★	★	★	★	7
22	Tuncel, D.	2009	★	★	★	★	★★	★	★	★	9
23	Menza, M.	2010	★	★			★★	★	★	★	7
24	Pålhagen, S.	2010	★	★			★★	★	★	★	7
25	Scalzo, P.	2010		★	★	★	★★	★	★	★	8
26	Varani, K.	2010	★	★	★	★	★	★	★	★	8
27	Hassin-Baer, S.	2011	★	★			★★	★	★	★	7
28	Lee, H. W.	2011	★	★		★	★★	★	★	★	8
29	Scalzo, P.	2011		★		★	★★	★	★	★	7
30	Shi, M.	2011	★	★	★	★	★	★	★	★	8
31	Andican, G.	2012		★		★	★★	★	★	★	7
32	Gruden, M. A.	2012		★		★	★	★	★	★	6
33	Koziorowski, D.	2012	★	★	★	★	★	★	★	★	8
34	Lindqvist, D.	2012		★	★	★	★★	★	★	★	8
35	Liu, J.	2012	★	★	★	★	★	★	★	★	8
36	Mahlknecht, P.	2012	★	★	★	★	★	★	★	★	8
37	Sathe, K.	2012	★	★		★	★	★	★	★	7
38	Ton, T. G.	2012		★	★	★		★	★	★	6
39	Zhao, X. Q.	2012	★	★	★	★	★★	★	★	★	9
40	Lindqvist, D.	2013		★	★	★	★★	★	★	★	8
41	Olsson, B.	2013	★	★		★	★	★	★	★	7

42	Rocha, N. P.	2014	★	★	★	★	★★	★	★	★	9
43	Rocha, N. P.	2014	★	★	★	★	★★	★	★	★	9
44	Sawada, H.	2014	★	★	★	★	★★	★	★	★	9
45	Song, I. U.	2014	★	★		★	★	★	★	★	7
46	Tang, P.	2014	★	★	★	★	★	★	★	★	8
47	Wang, Y. R.	2014	★	★		★	★★	★	★	★	8
48	Yu, S. Y.	2014	★	★	★	★	★	★	★	★	8
49	Akıl, E.	2015	★	★	★	★	★	★	★	★	8
50	Bu, X. L.	2015	★	★		★	★	★	★	★	7
51	Hu, Y.	2015	★	★		★	★	★	★	★	7
52	Jiang, Q. W.	2015	★	★	★	★	★	★	★	★	8
53	Magdalinou, N. K.	2015		★	★	★	★	★	★	★	7
54	Martín de Pablos, A.	2015	★	★	★	★	★	★	★	★	8
55	Park, S. J.	2015	★	★	★	★	★★	★	★	★	9
56	Umemura, A.	2015	★	★		★	★★	★	★	★	8
57	Wang, Y. H.	2015	★	★		★	★	★	★	★	7
58	Wennstrom, M.	2015	★	★	★	★	★	★	★	★	8
59	Csencsits-Smith, K.	2016	★	★		★	★	★	★	★	7
60	da Silva, D. J.	2016	★	★		★	★	★	★	★	7
61	de Farias, C. C.	2016	★	★	★	★	★	★	★	★	8
62	Gupta, V.	2016	★	★		★	★	★	★	★	7
63	Hall, S.	2016		★	★	★	★	★	★	★	7
64	Li, Z. J.	2016	★	★	★	★	★	★	★	★	8
65	Lue, L. F.	2016	★	★	★	★	★★	★	★	★	9
66	Pereira, J. R.	2016	★	★	★	★	★★	★	★	★	9
67	Wang, X. M.	2016	★	★	★	★	★	★	★	★	8
68	Williams-Gray, C. H.	2016	★	★	★	★	★	★	★	★	8
69	Delgado-Alvarado, M.	2017	★	★	★	★		★	★	★	7
70	Eidson, L. N.	2017	★	★		★	★	★	★	★	7
71	Bagheri, V.	2018	★	★	★	★	★★	★	★	★	9
72	Dos Santos, M. C. T.	2018	★	★		★	★	★	★	★	7
73	Hall, S.	2018	★	★	★	★	★★	★	★	★	9
74	Karpenko, M. N.	2018		★	★	★	★★	★	★	★	8
75	Kim, R.	2018	★	★	★	★	★★	★	★	★	9
76	Kouchaki, E.	2018	★	★	★	★	★	★	★	★	8
77	Kouchaki, E.	2018	★	★	★	★	★	★	★	★	8
78	Ilyechova, E. Y.	2018	★	★	★	★	★	★	★	★	8
79	Li, D. H.	2018	★	★	★	★	★★	★	★	★	9
80	Moghaddam, H. S.	2018		★	★	★	★	★	★	★	7
81	Pochmann, D.	2018		★		★	★	★	★	★	6
82	Rocha, N. P.	2018	★	★	★	★	★★	★	★	★	9
83	Rocha Sobrinho, H. M. D.	2018	★	★	★	★	★★	★	★	★	9
84	Solmaz, V.	2018	★	★		★	★	★	★	★	7
85	Starhof, C.	2018		★		★	★	★	★	★	6

86	Veselý, B.	2018	★	★			★	★	★	★	6
87	Yang, Y.	2018	★	★	★	★	★★	★	★	★	9
88	Ahmadi Rastegar, D.	2019	★	★			★★	★	★	★	7
89	Alrafiah, A.	2019		★	★	★	★	★	★	★	7
90	Álvarez-Luquín, D. D.	2019	★	★		★	★	★	★	★	7
91	Baran, A.	2019	★	★	★	★	★★	★	★	★	9
92	Compta, Y.	2019		★	★	★	★	★	★	★	7
93	Green, H. F.	2019		★		★	★	★	★	★	6
94	King, E.	2019	★	★	★	★	★	★	★	★	8
95	Lian, T. H.	2019	★	★		★	★★	★	★	★	8
96	Lin, C. H.	2019	★	★	★	★	★★	★	★	★	9
97	Mollenhauer, B.	2019	★	★		★	★	★	★	★	7
98	Perner, C.	2019	★	★		★	★★	★	★	★	8
99	Santos-Garcia, D.	2019		★		★	★★	★	★	★	7
100	Yazar, T.	2019	★	★		★	★	★	★	★	7
101	Calvani, R.	2020	★	★		★	★	★	★	★	7
102	Chatterjee, K.	2020	★	★	★	★	★★	★	★	★	9
103	Fan. Z.	2020	★	★	★	★	★★	★	★	★	9
104	Kiçik, A.	2020	★	★	★	★	★★	★	★	★	9
105	Iwaoka, K.	2020	★	★		★	★★	★	★	★	8
106	Jin, H.	2020	★	★	★	★	★★	★	★	★	9
107	Kwiatek-Majkusiak, J.	2020	★	★	★	★	★	★	★	★	8
108	Lian, T. H.	2020	★	★	★	★	★★	★	★	★	9
109	Majbour, N. K.	2020	★	★	★	★	★	★	★	★	8
110	Martin-Ruiz, C.	2020	★	★		★	★	★	★	★	7
111	Miliukhina, I. V.	2020	★	★	★	★	★	★	★	★	8
112	Moghadam-Ahmadi, A.	2020	★	★	★	★	★	★	★	★	8
113	Peng, G.	2020	★	★		★	★	★	★	★	7
114	Sankowski, B.	2020	★	★	★	★	★	★	★	★	8
115	Santaella, A.	2020	★	★	★	★	★★	★	★	★	9
116	Santaella, A.	2020	★	★	★	★	★★	★	★	★	9
117	Szymura, J.	2020		★		★	★	★	★	★	6
118	Usenko, T. S.	2020	★	★		★	★	★	★	★	7
119	Yang, W. L.	2020	★	★		★	★	★	★	★	7
120	Yu, C. C.	2020	★	★	★	★	★	★	★	★	8
121	Bartl, M.	2021	★	★	★	★	★	★	★	★	8
122	Chen, S. J.	2021	★	★		★	★	★	★	★	7
123	Galper, J.	2021		★		★	★	★	★	★	6
124	Hatcher-Martin, J. M.	2021	★	★	★	★	★	★	★	★	8
125	Hu, Y.	2021	★	★	★	★	★	★	★	★	8
126	Li, S. Y.	2021	★	★			★	★	★	★	6
127	Lin, Y. W.	2021	★	★	★	★	★	★	★	★	8
128	Mo, M. S.	2021	★	★	★	★	★	★	★	★	8
129	Muruzheva, Z. M.	2021	★	★	★	★	★	★	★	★	8

130	Roy, A.	2021		★		★	★	★	★	★	6
131	Solini, A.	2021	★	★	★	★	★	★	★	★	8
132	Thaler, A.	2021	★	★	★	★	★	★	★	★	8
133	Wu, H. C.	2021	★	★		★	★★	★	★	★	8
134	Wu, Z. B.	2021	★	★		★	★	★	★	★	7
135	Xia, D. H.	2021	★	★	★	★	★	★	★	★	8
136	Yin, Y. H.	2021	★	★	★	★	★★	★	★	★	9
137	Zhu, Y.	2021	★	★		★	★	★	★	★	7
138	Al-Huchaimi, S. H. K.	2022	★	★	★	★	★	★	★	★	8
139	Diaz, K.	2022		★		★	★	★	★	★	6
140	El-Kattan, M. M.	2022	★	★	★	★	★	★	★	★	8
141	Ghit, A.	2022	★	★	★	★	★	★	★	★	8
142	Gupta, M.	2022	★	★	★	★	★	★	★	★	8
143	Haji, S.	2022	★	★	★	★	★	★	★	★	8
144	Imarisio, A.	2022		★		★	★★	★	★	★	7
145	Kaminska, M.	2022	★	★	★	★	★★	★	★	★	9
146	Lerche, S.	2022	★	★			★	★	★	★	6
147	Li, Y. Y.	2022	★	★	★	★	★	★	★	★	8
148	Liu, J.	2022	★	★	★	★	★★	★	★	★	9
149	Wang, L. X.	2022	★	★		★	★★	★	★	★	8
150	Xu, J. H.	2022	★	★		★	★	★	★	★	7
151	Yuan, Y.	2022	★	★	★	★	★	★	★	★	8
152	Zhao, Y.	2022	★	★	★	★	★	★	★	★	8

Supplementary Table 5. Official cytokine names.

Cytokines	Official Names
BNDF	Brain-derived neurotrophic factor
CCL2 (MCP-1)	Chemokine (C-C motif) ligand 2; Monocyte chemoattractant protein-1
CCL3 (MIP-1α)	Chemokine (C-C motif) ligand 3; Monocyte chemoattractant protein-1 alpha
CCL4 (MIP-1β)	Chemokine (C-C motif) ligand 4; Monocyte chemoattractant protein-1 beta
CCL5 (RANTES)	Chemokine (C-C motif) ligand 5; Regulated upon activation normal T cell expressed and secreted factor
CCL7 (MCP-3)	Chemokine (C-C motif) ligand 3; Monocyte chemoattractant protein-3
CCL8 (MCP-2)	Chemokine (C-C motif) ligand 8; Monocyte chemoattractant protein-2
CCL11 (eotaxin-1)	Chemokine (C-C motif) ligand 11
CCL13 (MCP-4)	Monocyte chemotactic protein 13; Monocyte chemoattractant protein-4
CCL15 (MIP-5)	Chemokine (C-C motif) ligand 15; Macrophage inflammatory protein-5
CCL18 (MIP-4)	Chemokine (C-C motif) ligand 18; Macrophage inflammatory protein-4
CCL20 (MIP-3α)	Chemokine (C-C motif) ligand 20; Macrophage inflammatory protein-3 alpha
CCL23 (MIP-3)	Chemokine (C-C motif) ligand 23; Macrophage inflammatory protein-3
CCL24 (Eotaxin-2)	Chemokine (C-C motif) ligand 24
CCL28 (MEC)	Chemokine (C-C motif) ligand 28; Mucosae-associated epithelial chemokine
CRP	C-reactive protein
CSF-1	Macrophage-colony stimulating factor-1
CX3CL1 (Fractalkine)	CX3 chemokine ligand 1
CXCL1 (GRO-α)	Chemokine (C-X-C motif) ligand 1 protein; Growth regulated oncogene alpha
CXCL10 (IP-10)	Chemokine (C-X-C motif) ligand 10 protein; Interferon-γ-inducible protein 10
CXCL12 (SDF-1)	Chemokine (C-X-C motif) ligand 12 protein; Stromal cell-derived factor-1
EGF	Epidermal growth factor
FGF basic	Fibroblast growth factor-basic
FGF-19	Fibroblast growth factor-19
FGF-2	Fibroblast growth factor-2
Flt-3L	Fms related tyrosine kinase 3 ligand
G-CSF	Granulocyte colony-stimulating factor
GFAP	Glial fibrillary acidic protein
GM-CSF	Granulocyte macrophage-colony stimulating factor
GRO	Growth-regulated oncogene
H_2O_2	Hydrogen peroxide
HMGB1	High mobility group box 1
IFN-α2	Interferon-alpha 2
IFN-γ	Interferon-gamma
IGF-1	Insulin-like growth factor-1

IL-1	Interleukin-1
IL-1α	Interleukin-1 alpha
IL-1β	Interleukin-1 beta
IL-1RA	Interleukin-1 receptor antagonist
IL-2	Interleukin-2
IL-2R	IL-2 receptor
IL-3	Interleukin-3
IL-4	Interleukin-4
IL-5	Interleukin-5
IL-6	Interleukin-6
IL-7	Interleukin-7
IL-8	Interleukin-8
IL-9	Interleukin-9
IL-10	Interleukin-10
IL-12	Interleukin-12
IL-12 P40	IL-12 P40 chain
IL-12 P70	IL-12 P70 chain
IL-13	Interleukin-13
IL-16	Interleukin-16
IL-17A	Interleukin-17
IL-18	Interleukin-18
IL-21	Interleukin-21
IL-23	Interleukin-23
IL-27	Interleukin-27
IL-33	Interleukin-33
MDC	Monocyte-derived dendritic cell
NGAL	Neutrophil gelatinase-associated lipocalin
NLRP3	NOD-like receptor thermal protein domain associated protein 3
NO	Nitric oxide
NT-pro BNP	N-terminal pro-B-type natriuretic peptide
PD-L1	Programmed death-ligand 1
PDGF	Platelet-derived growth factor
PDGFB	Platelet-derived growth factor-B
PEG2	Prostaglandin E2
PTX3	Pentraxin 3
S100β	Central nervous system specific protein beta
SAA	Serum amyloid A protein
sAPP-α	Amyloid precursor protein-alpha
sAPP-β	Amyloid precursor protein-beta
sCD40L	Soluble CD40 ligand
SCF	Stem cell factor
sICAM-1	Soluble intercellular adhesion molecule-1
sLAG3	Soluble lymphocyte-activation gene 3
sTNFR1	Soluble tumor necrosis factor receptor 1

sTNFR2	Soluble tumor necrosis factor receptor 2
sTREM2	Soluble triggering receptor expressed on myeloid cells 2
sVCAM-1	Soluble vascular cell adhesion molecule-1
TGF-α	Transforming growth factor-alpha
TGF-β	Transforming growth factor-beta
TGF-β1	Transforming growth factor-beta1
TGF-β2	Transforming growth factor-beta2
TIPE2	Tumor necrosis factor -α induced protein 8 like -2
TNF-α	Tumor necrosis factor-alpha
TNF-β	Tumor necrosis factor-beta
VEGF-A	Vascular endothelial growth factor A
YKL-40	Chitinase protein 40; Chitinase-3-like protein1 (CHI3L1)
β-NGF	β-nerve growth factor

Supplementary Table 6. Summary of comparative outcomes of peripheral blood cytokines levels in individuals with PD versus controls.

Cytokines	No. Of Studies	No. Of Cases		Main Effect					Heterogeneity				Publication Bias	
		PD	HC	Hedges's g	LCI	UCI	z Score	P Value	Q Statistic	df	P Value	I^2 (%)	Egger Intercept	P Value
IL-6	44	2931	7912	0.603	0.325	0.881	4.25	0.000	940.9	43	0.000	95.4	4.787	0.009
TNF-α	40	2838	2121	0.593	0.293	0.894	3.871	0.000	898.3	39	0.000	95.7	2.843	0.207
CRP	34	3593	8191	0.510	0.313	0.706	5.09	0.000	503.3	33	0.000	93.4	2.733	0.047
IL-1β	23	1325	874	1.300	0.709	1.892	4.31	0.000	743.6	22	0.000	97.0	8.291	0.008
IL-10	19	954	680	0.028	-0.279	0.336	0.18	0.856	149.7	18	0.000	88.0	1.047	0.589
IFN-γ	17	993	927	-0.385	-0.743	-0.026	-2.10	0.035	210.7	16	0.000	92.4	-4.759	0.018
IL-8	11	569	485	-0.025	-0.683	0.632	-0.08	0.940	239.5	10	0.000	95.8	-1.758	0.702
IL-2	10	572	370	0.274	-0.249	0.797	1.03	0.304	115.8	9	0.000	92.2	4.012	0.200
IL-4	7	316	214	-0.710	-1.336	-0.084	-2.22	0.026	66.7	6	0.000	91.0	-0.613	0.897
MIP-1α	7	444	371	-0.356	-0.900	0.188	-1.28	0.199	81.7	6	0.000	92.7	-3.767	0.251
CCL2 (MCP-1)	6	216	191	0.911	0.246	1.576	2.69	0.007	36.2	5	0.000	86.2	1.520	0.667
IL-12	6	388	265	0.356	-0.513	1.225	0.80	0.422	96.4	5	0.000	94.8	7.257	0.023
CCL11 (Eotaxin)	6	224	183	0.167	-0.445	0.779	0.54	0.592	63.2	5	0.000	92.1	5.908	0.159
NO	5	634	231	-0.040	-0.698	0.617	-0.12	0.904	61.2	4	0.000	93.5	9.643	0.073
IL-13	5	390	336	0.545	-0.732	1.821	0.84	0.403	196.2	4	0.000	98.0	4.457	0.590
CXCL10 (IP-10)	5	329	236	0.439	-0.132	1.009	1.51	0.132	45.2	4	0.000	91.2	6.131	0.002
IL-17A	5	561	367	0.368	-0.262	0.999	1.14	0.252	66.5	4	0.000	94.0	1.956	0.685
CCL4 (MIP-1β)	4	178	176	0.158	-1.000	1.316	0.27	0.789	73.2	3	0.000	95.9	10.032	0.294
CX3CL1	4	209	199	0.361	0.166	0.556	3.63	0.000	1.0	3	0.813	0.0	-1.418	0.212

(Fractalkine)														
IL-1RA	4	363	249	-1.189	-2.641	0.263	-1.61	0.108	121.0	3	0.000	97.5	-4.160	0.611
IL-9	4	326	225	0.647	-0.836	2.131	0.86	0.392	112.7	3	0.000	97.3	7.513	0.293
CCL5 (RANTES）	4	224	181	0.016	-1.060	1.092	0.03	0.977	51.8	3	0.000	94.2	-4.961	0.273
IL-1	4	259	164	1.230	-0.424	2.884	1.46	0.145	127.3	3	0.000	97.6	8.565	0.456
IL-2R	4	265	189	0.283	-0.302	0.867	0.95	0.343	25.7	3	0.000	88.3	-4.836	0.568
STNFR1	4	210	243	0.449	0.004	0.894	1.98	0.048	12.8	3	0.005	76.6	5.833	0.016
STNFR2	4	210	243	0.157	-0.120	0.434	1.11	0.266	5.3	3	0.151	43.4	3.661	0.063
PEG2	3	192	96	0.044	-0.593	0.681	0.14	0.892	12.2	2	0.002	83.6	14.486	0.305
H_2O_2	3	404	96	0.062	-0.340	0.465	0.30	0.761	6.5	2	0.038	69.3	-91.998	0.367
IL-12 P70	3	44	46	-0.080	-0.494	0.335	-0.38	0.707	1.8	2	0.409	0.0	-1.584	0.846
IL-12 P40	3	42	43	0.222	-0.461	0.905	0.64	0.524	4.8	2	0.090	58.5	0.775	0.947
YKL-40	3	115	114	1.523	-0.708	3.754	1.34	0.181	98.6	2	0.000	98.0	20.200	0.064
IL-7	3	242	188	-0.401	-1.404	0.602	-0.78	0.433	19.8	2	0.000	89.9	4.146	0.353
CXCL12 (SDF-1)	3	186	192	2.933	0.883	4.983	2.80	0.005	120.5	2	0.000	98.3	13.726	0.199
IGF-1	3	186	192	0.534	0.355	0.714	5.83	0.000	2.2	2	0.329	10.0	13.726	0.199
CCL24 (Eotaxin-2)	3	117	78	0.609	-0.086	1.303	1.72	0.086	21.9	2	0.000	90.9	9.995	0.039
sICAM-1	3	175	121	0.194	-0.488	0.877	0.56	0.576	14.8	2	0.001	86.5	5.890	0.466
IL-1A	2	32	28	-0.096	-0.603	0.411	-0.37	0.710	0.16	1	0.685	0.0	-	-
TGF-β	2	131	112	-0.497	-1.917	0.922	-0.69	0.492	26.3	1	0.000	96.2	-	-
TGF-β1	2	43	57	-0.202	-1.184	0.780	-0.40	0.687	5.6	1	0.018	82.3	-	-
G-CSF	2	99	92	-0.830	-2.226	0.566	-1.17	0.244	14.7	1	0.000	93.2	-	-
IL-5	2	67	73	0.023	-0.788	0.834	0.06	0.956	2.3	1	0.126	57.3	-	-

IFN-α2	2	44	34	-0.831	-1.444	-0.219	-2.66	0.008	1.8	1	0.185	43.0	-	-
NLPR3	2	47	30	3.506	-1.822	8.835	1.29	0.197	46.8	1	0.000	97.9	-	-
S100β	2	235	129	-0.041	-0.483	0.401	-0.18	0.854	4.1	1	0.043	75.6	-	-
CCL15 (MIP-5)	2	156	152	-0.204	-0.427	0.019	-1.80	0.073	0.6	1	0.436	0.0	-	-
IL-21	2	101	48	1.166	-1.376	3.708	0.90	0.369	40.8	1	0.000	97.5	-	-
NT-pro BNP	2	192	70	0.533	0.256	0.809	3.78	0.000	0.7	1	0.415	0.0	-	-
sLAG3	2	66	57	0.262	-0.949	1.473	0.42	0.672	9.1	1	0.003	89.0	-	-

Supplementary Table 7. Summary of comparative outcomes of cerebrospinal fluid cytokines levels in individuals with PD versus controls.

Cytokines	No. Of Studies	No. Of Cases		Main Effect					Heterogeneity				Publication Bias	
		PD	HC	Hedges's g	LCI	UCI	z Score	P Value	Q Statistic	df	P Value	I^2 (%)	Egger Intercept	P Value
IL-6	14	816	440	0.559	0.163	0.955	2.76	0.006	126.0	13	0.000	89.7	4.218	0.027
TNF-α	11	448	280	0.599	0.023	1.175	2.04	0.042	123.0	10	0.000	91.9	8.160	0.002
IL-1β	11	444	252	0.326	0.105	0.547	2.89	0.004	194.7	7	0.000	96.4	3.105	0.083
CRP	8	473	303	1.231	0.321	2.141	2.65	0.008	19.9	10	0.030	49.7	7.361	0.080
IFN-γ	6	152	123	-0.407	-1.100	0.287	-1.15	0.250	5.4	3	0.143	44.7	-3.185	0.463
CCL2 (MCP-1)	5	258	141	0.351	0.090	0.612	2.64	0.008	36.4	5	0.000	86.3	0.354	0.736
IL-10	4	113	100	-0.003	-0.376	0.370	-0.02	0.988	23.8	3	0.000	87.4	4.523	0.336
IL-8	4	206	96	-0.208	-0.999	0.583	-0.51	0.607	24.3	1	0.000	95.9	-3.743	0.432
IL-4	4	109	85	0.024	-0.834	0.883	0.05	0.956	5.5	4	0.240	27.3	5.784	0.389
NO	4	268	112	0.901	0.188	1.614	2.48	0.013	23.7	3	0.000	87.3	8.708	0.022
PEG2	4	488	127	-0.111	-0.460	0.238	-0.62	0.533	25.5	3	0.000	88.2	-24.570	0.499
TGF-β1	4	127	93	1.456	-0.098	3.011	1.84	0.066	9.5	3	0.023	68.4	15.453	0.109
CX3CL1 (Fractalkine)	4	208	187	-0.454	-1.193	0.286	-1.20	0.229	4.0	2	0.134	50.3	-4.329	0.223
YKL-40	4	464	232	-0.222	-0.748	0.305	-0.83	0.409	0.2	1	0.623	0.0	1.718	0.785
S100β	4	444	289	0.276	-0.063	0.614	1.60	0.110	0.2	1	0.625	0.0	4.829	0.513
H_2O_2	3	250	96	0.164	-0.172	0.500	0.95	0.340	0.0	0	1.000	0.0	-15.699	0.370
TGF-α	3	82	53	0.442	-0.809	1.693	0.69	0.489	0.3	1	0.602	0.0	12.527	0.020
IL-2	2	33	26	1.358	-1.811	4.527	0.84	0.401	0.0	0	1.000	0.0	-	-
IL-12 P70	2	32	38	-0.109	-0.581	0.363	-0.45	0.651	69.7	3	0.000	95.7	-	-
IL-12 P40	2	24	27	-0.039	-0.604	0.525	-0.14	0.891	55.9	1	0.000	98.2	-	-
CCL4	2	86	45	-0.266	-0.626	0.094	-1.45	0.147	25.5	3	0.000	88.3	-	-

(MIP-1β)														
TGF-β2	2	59	37	2.647	-2.379	7.673	1.03	0.302	0.0	0	1.000	0.0	-	-
CCL11 (Eotaxin）	2	85	44	0.265	-0.098	0.628	1.43	0.152	0.0	0	1.000	0.0	-	-
IL-18	2	80	60	0.059	-0.273	0.391	0.35	0.727	26.5	3	0.000	88.7	-	-
EGF	2	32	23	0.364	-0.807	1.535	0.61	0.542	0.1	1	0.794	0.0	-	-
sTREM2	2	332	180	0.073	-0.429	0.574	0.28	0.776	0.0	0	1.000	0.0	-	-

Supplementary Table 1. PRISMA 2009 Checklist.

Section/topic	#	Checklist item	Reported on page #
**TITLE**			
Title	1	Identify the report as a systematic review, meta-analysis, or both.	1
**ABSTRACT**			
Structured summary	2	Provide a structured summary including, as applicable: background; objectives; data sources; study eligibility criteria, participants, and interventions; study appraisal and synthesis methods; results; limitations; conclusions and implications of key findings; systematic review registration number.	2
**INTRODUCTION**			
Rationale	3	Describe the rationale for the review in the context of what is already known.	3
Objectives	4	Provide an explicit statement of questions being addressed with reference to participants, interventions, comparisons, outcomes, and study design (PICOS).	3-4
**METHODS**			
Protocol and registration	5	Indicate if a review protocol exists, if and where it can be accessed (e.g., Web address), and, if available, provide registration information including registration number.	4
Eligibility criteria	6	Specify study characteristics (e.g., PICOS, length of follow-up) and report characteristics (e.g., years considered, language, publication status) used as criteria for eligibility, giving rationale.	4
Information sources	7	Describe all information sources (e.g., databases with dates of coverage, contact with study authors to identify additional studies) in the search and date last searched.	4

Search	8	Present full electronic search strategy for at least one database, including any limits used, such that it could be repeated.	4
Study selection	9	State the process for selecting studies (i.e., screening, eligibility, included in systematic review, and, if applicable, included in the meta-analysis).	4
Data collection process	10	Describe method of data extraction from reports (e.g., piloted forms, independently, in duplicate) and any processes for obtaining and confirming data from investigators.	4-5
Data items	11	List and define all variables for which data were sought (e.g., PICOS, funding sources) and any assumptions and simplifications made.	4-5
Risk of bias in individual studies	12	Describe methods used for assessing risk of bias of individual studies (including specification of whether this was done at the study or outcome level), and how this information is to be used in any data synthesis.	5
Summary measures	13	State the principal summary measures (e.g., risk ratio, difference in means).	5
Synthesis of results	14	Describe the methods of handling data and combining results of studies, if done, including measures of consistency (e.g., I^2 for each meta-analysis.	
Risk of bias across studies	15	Specify any assessment of risk of bias that may affect the cumulative evidence (e.g., publication bias, selective reporting within studies).	5
Additional analyses	16	Describe methods of additional analyses (e.g., sensitivity or subgroup analyses, meta-regression), if done, indicating which were pre-specified.	5
**RESULTS**			
Study selection	17	Give numbers of studies screened, assessed for eligibility, and included in the review, with reasons for exclusions at each stage, ideally with a flow diagram.	6

Study characteristics	18	For each study, present characteristics for which data were extracted (e.g., study size, PICOS, follow-up period) and provide the citations.	6
Risk of bias within studies	19	Present data on risk of bias of each study and, if available, any outcome level assessment (see item 12).	7-8
Results of individual studies	20	For all outcomes considered (benefits or harms), present, for each study: (a) simple summary data for each intervention group (b) effect estimates and confidence intervals, ideally with a forest plot.	6-8
Synthesis of results	21	Present results of each meta-analysis done, including confidence intervals and measures of consistency.	6-8
Risk of bias across studies	22	Present results of any assessment of risk of bias across studies (see Item 15).	7-8
Additional analysis	23	Give results of additional analyses, if done (e.g., sensitivity or subgroup analyses, meta-regression [see Item 16]).	7-8
**DISCUSSION**			
Summary of evidence	24	Summarize the main findings including the strength of evidence for each main outcome; consider their relevance to key groups (e.g., healthcare providers, users, and policy makers).	8-9
Limitations	25	Discuss limitations at study and outcome level (e.g., risk of bias), and at review-level (e.g., incomplete retrieval of identified research, reporting bias).	9-13
Conclusions	26	Provide a general interpretation of the results in the context of other evidence, and implications for future research.	13
**FUNDING**			
Funding	27	Describe sources of funding for the systematic review and other support (e.g., supply of data); role of funders for the systematic review.	13

From: Moher D, Liberati A, Tetzlaff J, Altman DG, The PRISMA Group (2009). Preferred Reporting Items for Systematic Reviews and Meta-Analyses: The PRISMA Statement. PLoS Med 6(7): e1000097. doi:10.1371/journal.pmed1000097

Supplementary Table 2. Search strategy.

MeSH	Terms
Parkinson's disease	'Parkinson disease' OR Parkinson OR Parkinsonism
Inflammation	Inflammation OR inflammatory OR 'Tumor Necrosis Factor' OR Cytokines OR 'C-reactive protein' OR interleukin OR chemokine OR interferon OR 'transforming growth factor'
Sources	Peripheral OR CSF OR 'cerebrospinal fluid' OR blood OR serum OR plasma
Combined	('Parkinson disease' OR Parkinson OR Parkinsonism) AND (Inflammation OR inflammatory OR 'Tumor Necrosis Factor' OR Cytokines OR 'C-reactive protein' OR interleukin OR chemokine OR interferon OR 'transforming growth factor') AND (peripheral OR CSF OR 'cerebrospinal fluid' OR blood OR serum OR plasma)

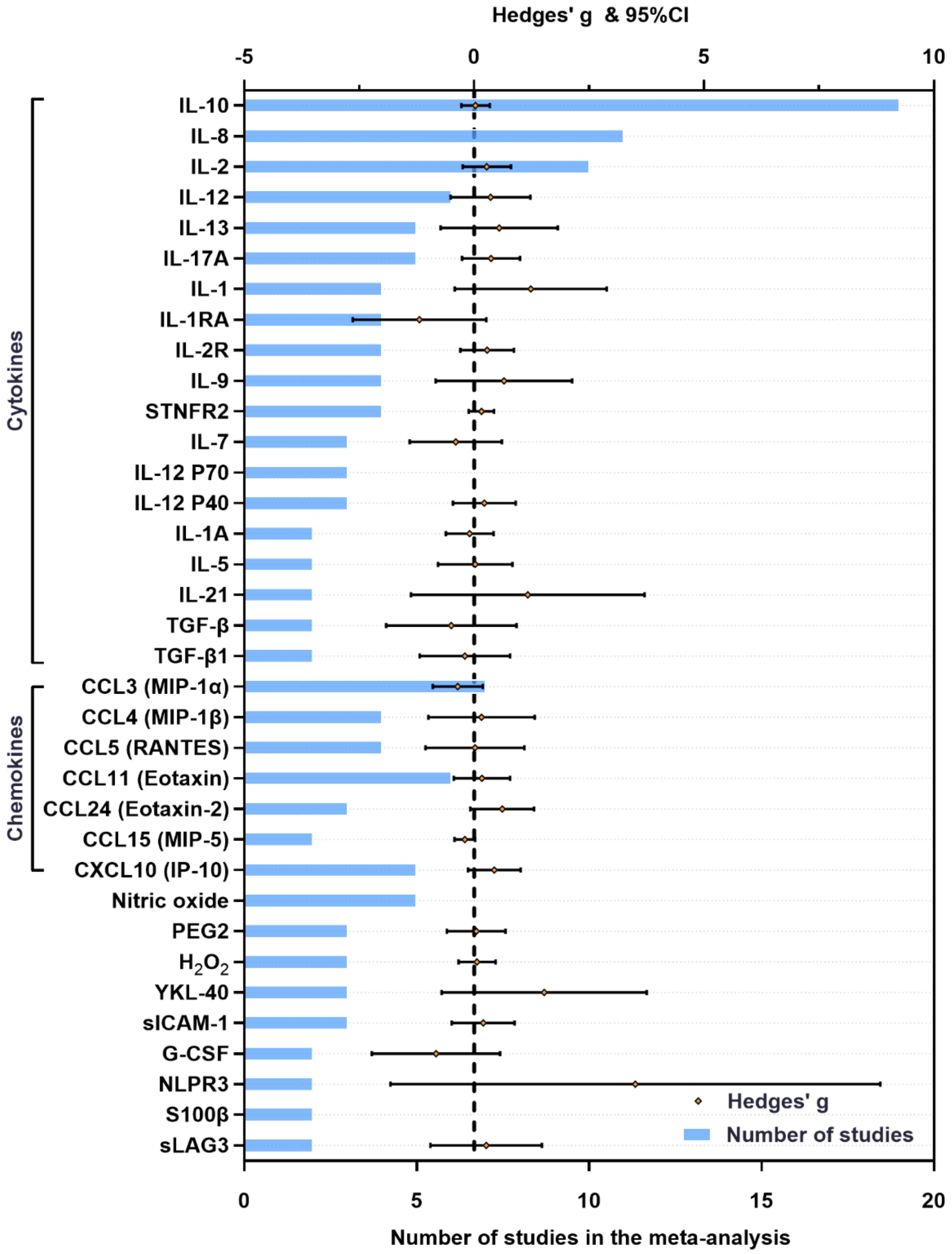

Supplementary Figure 1. Comparative outcomes of peripheral blood cytokines levels for PD patients versus controls (not significant).

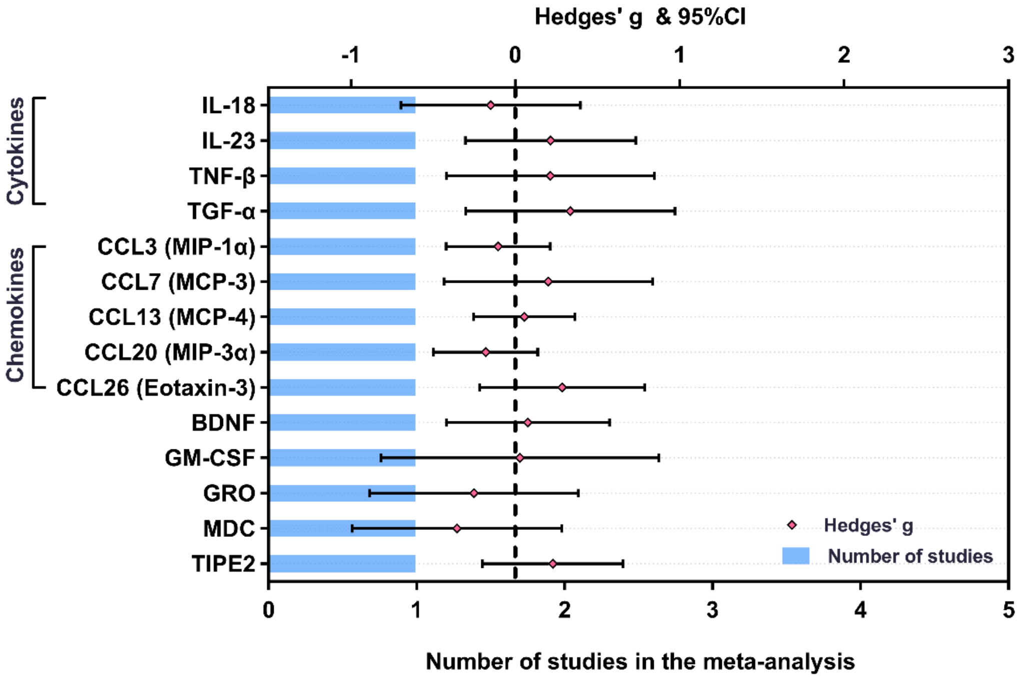

Supplementary Figure 2. Comparative outcomes of peripheral blood cytokines levels for PD patients versus controls in the systematic review (not significant).

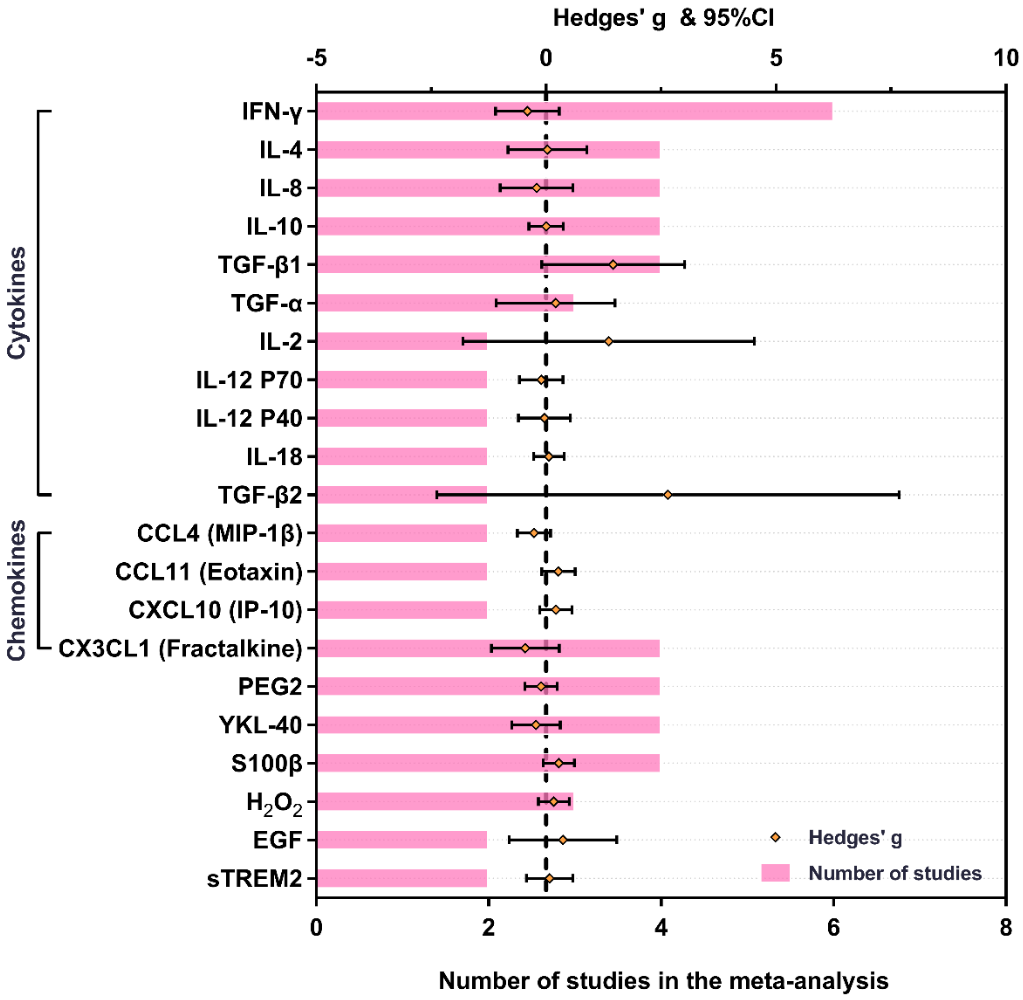

Supplementary Figure 3. Comparative outcomes of cerebrospinal fluid cytokines levels for PD patients versus controls (not significant).

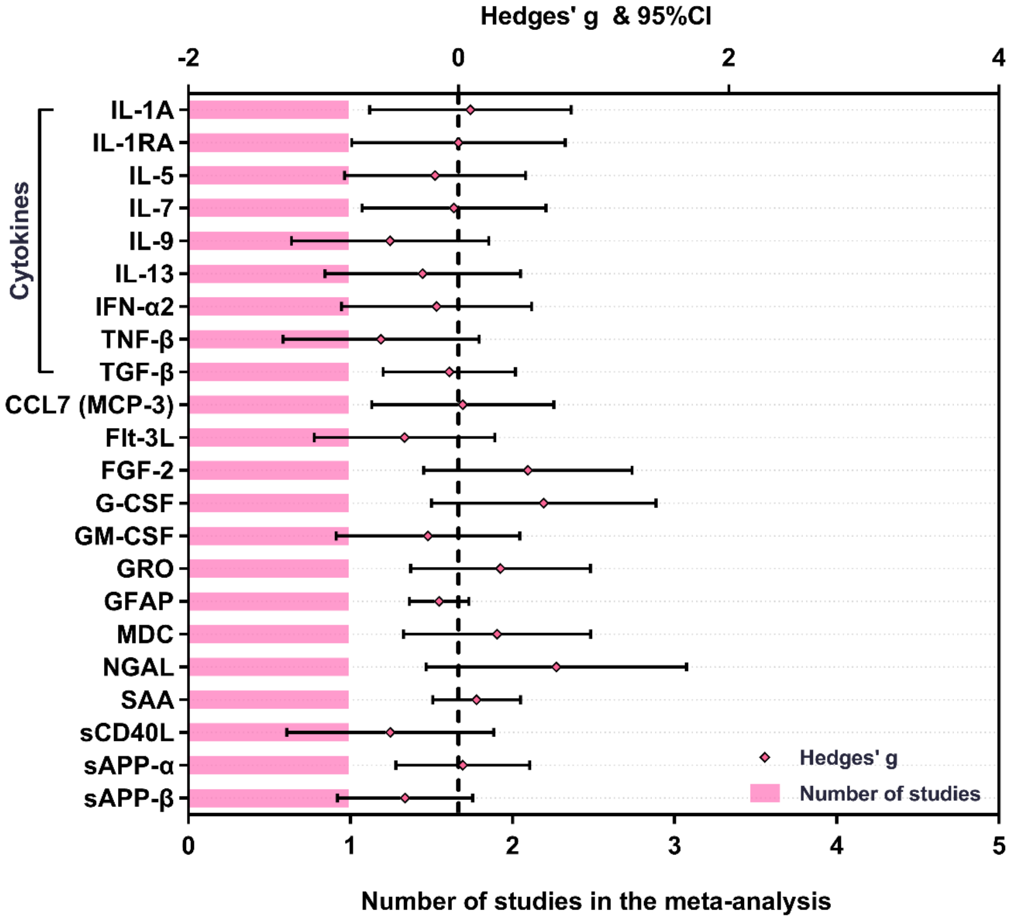

Supplementary Figure 4. Comparative outcomes of cerebrospinal fluid cytokines levels for PD patients versus controls in the systematic review (not significant).

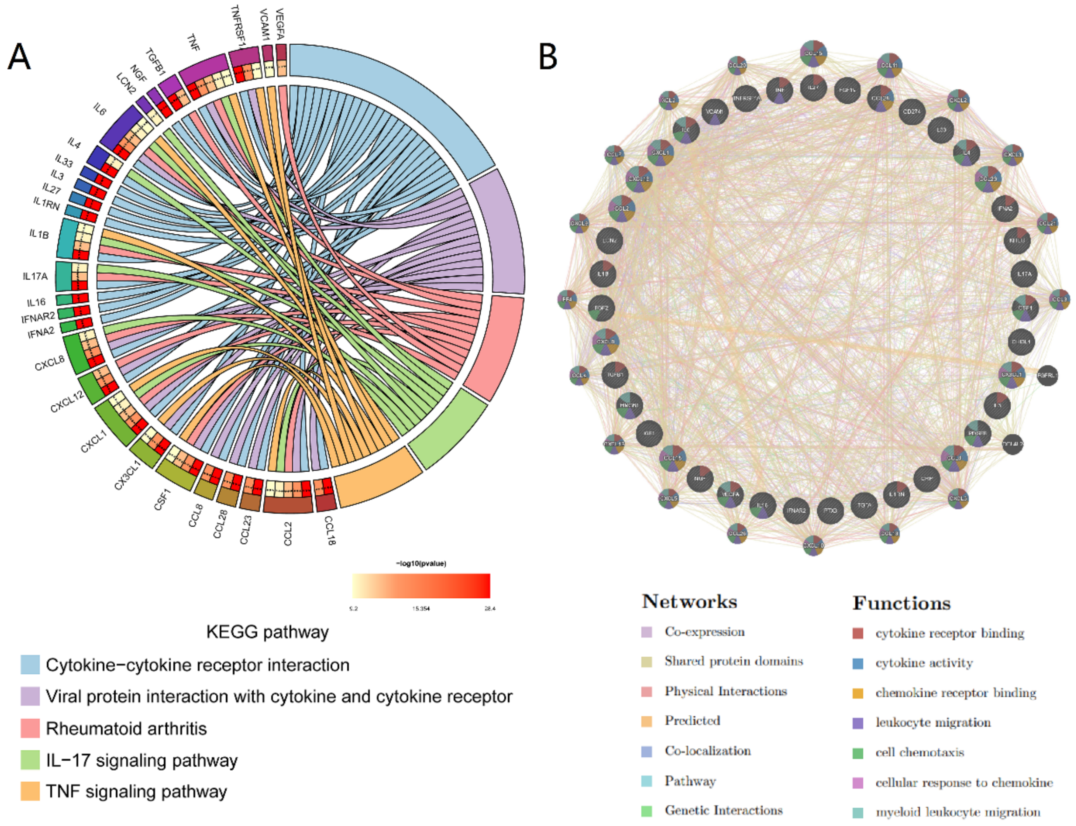

Supplementary Figure 5. KEGG pathway enrichment analysis (A) and PPI network construction analysis (B).
